# Rare variants in pharmacogenes influence clozapine metabolism in individuals with schizophrenia

**DOI:** 10.1016/j.euroneuro.2023.12.007

**Published:** 2024-02-03

**Authors:** Djenifer B. Kappel, Elliott Rees, Eilidh Fenner, Adrian King, John Jansen, Marinka Helthuis, Michael J. Owen, Michael C. O’Donovan, James T.R. Walters, Antonio F. Pardiñas

**Affiliations:** a Centre for Neuropsychiatric Genetics and Genomics, Division of Psychological Medicine and Clinical Neurosciences, School of Medicine, Cardiff University, Cardiff, United Kingdom; b Magna Laboratories Ltd., Ross-on-Wye, United Kingdom; c Leyden Delta B.V., Nijmegen, The Netherlands

**Keywords:** Pharmacogenomics, Schizophrenia, Clozapine, Metabolism, Whole exome sequencing

## Abstract

Clozapine is the only licensed medication for treatment-resistant schizophrenia (TRS). Few predictors for variation in response to clozapine have been identified, but clozapine metabolism is known to influence therapeutic response and adverse side effects. Here, we expand on genome-wide studies of clozapine metabolism, previously focused on common genetic variation, by analysing whole-exome sequencing data from 2062 individuals with schizophrenia taking clozapine in the UK. We investigated whether rare genomic variation in genes and gene sets involved in the clozapine metabolism pathway influences plasma concentrations of clozapine metabolites, assessed through the longitudinal analysis of 6585 pharmacokinetic assays. We observed a statistically significant association between the burden of rare damaging coding variants (MAF ≤ 1 %) in gene sets broadly related to drug pharmacokinetics and lower clozapine (β = − 0.054, SE = 0.019, *P*-value = 0.005) concentrations in plasma. We estimate that the effects in clozapine plasma concentrations of a single damaging allele in this gene set are akin to reducing the clozapine dose by about 35 mg/day. The gene-based analysis identified rare variants in *CYP1A2*, which encodes the enzyme responsible for converting clozapine to norclozapine, as having the strongest effects of any gene on clozapine metabolism (β = 0.324, SE = 0.124, *P* = 0.009). Our findings support the hypothesis that rare genetic variants in known drug-metabolising enzymes and transporters can markedly influence clozapine plasma concentrations; these results suggest that pharmacogenomic efforts trying to predict clozapine metabolism and personalise drug therapy could benefit from the inclusion of rare damaging variants in pharmacogenes beyond those already identified and catalogued as PGx star alleles.

## 1 Introduction

Schizophrenia is a chronic psychiatric disorder affecting 7 out of 1000 people worldwide (McGrath et al., 2008). Between 20–30 % of individuals with schizophrenia develop treatment-resistant schizophrenia (TRS), a particularly severe presentation of the disorder in which symptoms do not respond to treatment after at least two courses of standard antipsychotics (Kane et al., 2019; Siskind et al., 2022). Clozapine is a second-generation antipsychotic considered the evidence-based treatment of choice for TRS, with good efficacy in targeting refractory symptoms and better outcomes when compared with other pharmacological treatments (Wagner et al., 2021). Clinical guidelines recommend clozapine initiation for TRS patients at the earliest possible opportunity, but the considerable risk of drug-induced adverse effects and toxicity hamper the use of clozapine in clinical practice, despite its apparent benefits (Wagner et al., 2021). In particular, due to the risk of agranulocytosis, a haematological adverse drug reaction (ADR), individuals treated with clozapine undergo regular mandatory blood monitoring. Therapeutic drug monitoring (TDM) frameworks have also been recommended to ensure clozapine dosing leads to plasma concentrations within the standard “therapeutic range” (350–600 ng/mL) that aims to maximise safety and treatment response and minimise the risk of drug-induced adverse effects and toxicity (Flanagan et al., 2023a; Siskind et al., 2021). Still, considerable inter-individual variability in drug pharmacokinetic processes such as absorption, distribution, metabolism, and excretion (ADME) can further influence psychiatric medication tolerance and effectiveness (Hiemke et al., 2018). It is estimated that a genetic component might explain 20–30 % of this variability (Kalow et al., 1998), and a better understanding of the specific genetic mechanisms linking clozapine metabolism, response, and toxicity may pave the way for safer and more efficient use of the drug in TRS clinical management.

Pharmacogenomic (PGx) research focuses on studying how genetic variation influences the response to drugs, with the aim of using genetically derived information to deliver personalised care. While previous research has indicated that 40 % of the inter-individual variability in clozapine metabolism is related to demographic, lifestyle and clinical predictors (sex, age, body weight and smoking) (Flanagan et al., 2023b), the few genetic studies performed to date have also found a small number of common genetic variants explaining up to an extra 3 %–8 % of variance (Pardiñas et al., 2023). As the effects of these genetic markers can be additive to those of environmental predictors (Lenk et al., 2022), their discovery is a first step towards eventual pre-emptive PGx interventions to personalise and improve clozapine treatment protocols.

Most PGx studies of clozapine and other psychiatric medications have been based on the “candidate gene” experimental design, investigating well-characterized polymorphisms and haplotypes in genes encoding cytochrome P450 enzymes (CYPs), commonly known as “star alleles” (Ahmed et al., 2016). These PGx markers are routinely assessed by genetic testing companies and, given their functional effects on enzyme activity (Bousman et al., 2021), have been proposed as useful to guide drug selection and dosing. The hepatic first-pass metabolism of clozapine is mainly driven by the CYP1A2 enzyme (with a minor role played by CYP2C19, CYP3A4, and CYP2D6), and therefore lifestyle factors and concomitant medications influencing the activity of this enzyme are commonly discussed in the literature (de Leon et al., 2022; Flanagan et al., 2023a). It has also been suggested that differences in clozapine metabolism can arise from the presence of variants in these and other genes involved in its metabolic processes; however, a clear relationship between *CYP1A2* genetic variation and clozapine metabolism has not yet been established, and no genetic variants are considered relevant for clinical management on recent expert reviews (Correll et al., 2022; de Leon et al., 2022) or in the PharmGKB database (Whirl-Carrillo et al., 2021). Evidence from previous genome-wide association studies (GWAS) suggests that clozapine metabolic traits are likely to be influenced by a small number of variants with a relatively large effect and could be considered to have an oligogenic genetic architecture (Pardiñas et al., 2019; Smith et al., 2020). These studies have also not implicated variants within the *CYP1A2* gene, although a 13 kb upstream variant potentially involved in its regulation, rs2472297, has a reported and replicated association (Lenk et al., 2022; Pardiñas et al., 2019).

It is important to consider that while both GWAS and candidate gene studies are best suited to identify the influences of relatively common alleles, rare variants also contribute to differences in medication-related traits and sequencing-based methods are required to detect this source of genetic variation. Indeed, analysis of exonic and genomic data have implicated rare variants in clozapine ADRs (Legge et al., 2017; Narang et al., 2022), though genes specifically involved in drug pharmacokinetics (“pharmacogenes”) have not been the focus of those studies. Pharmacogenes are commonly under low evolutionary pressure, accumulating a large number of genetic variants, of which the majority are rare (MAF < 1 %) or very rare (MAF < 0.1 %). Some of these variants have strong effects on metabolic phenotypes by altering the expression, the structure, or the enzyme function (Ingelman-Sundberg et al., 2018; Zhou et al., 2019), and have been found to be responsible for 30–40 % of the known functional variability of drug-related enzymes and transporter pharmacogenes (Ingelman-Sundberg et al., 2018; Wang et al., 2018; Zhou and Lauschke, 2022). The presence of rare variants with a functional effect on pharmacogenes involved in the clozapine metabolic pathway may contribute to the unexplained variability in drug metabolism and ultimately affect medication response, tolerability, and other clinical outcomes (Pardiñas et al., 2021; Thorn et al., 2018). Owing to the complexity of optimal clozapine prescription and the need for careful TDM, the identification of rare pharmacogenomic markers could facilitate the management of clozapine treatment in those with an atypical metabolism unrelated to known common variants or environmental influences. In this study, we build on our previous findings by assessing a different class of genetic variation, rare deleterious coding variants obtained from whole exome sequencing (WES) data from over 2000 individuals taking clozapine. Our aim was to investigate for the first time, whether rare variants (MAF ≤ 1 %) in genes involved in the clozapine metabolism pathway affect clozapine pharmacokinetics, further explaining the differences in clozapine plasma concentrations between individuals.

## 2 Experimental procedures

### Participants

Participants were from the CLOZUK2 study (Pardiñas et al., 2018), which received UK National Research Ethics Service approval (ref. 10/WSE02/15) in accordance with the UK Human Tissue Act. As previously reported (Pardiñas et al., 2018; Pardiñas et al., 2019), CLOZUK2 includes clozapine monitoring records from the Zaponex® Treatment Access System (ZTAS) and genetic information from anonymised individuals with TRS taking clozapine in the United Kingdom. Pharmacokinetic data derived from the monitoring records consisted of clozapine and norclozapine plasma concentrations (also known as “levels”) retrieved during 2013–2015. Briefly, data curation was performed as described in another study (Pardiñas et al., 2019), ensuring that all pharmacokinetic assays included in our analyses adhered to the following criteria: the individual was at least 18 years old and taking clozapine doses ≤900 mg/day; blood was drawn at an interval of 6 to 24 h after their last clozapine dose, and clozapine concentrations did not exceed 2000 ng/mL. The final curated dataset contained 3578 individuals linked to 11407 pharmacokinetic assays.

### Whole exome sequencing

Samples were prepared for whole exome sequencing (WES) using the Nextera® DNA Exome capture kit according to the manufacturer’s protocol. Once prepared, the exome-captured library was then sequenced in the Illumina HiSeq 3000/4000 platform (Illumina Inc, California) using the paired-end method, as previously described (Rees et al., 2020). Exome sequences had a median of 83 % of all targeted bases covered at ≥10×, raw data was processed to remove adaptors and low-quality reads, then aligned to the GRCh37 human reference genome with Burrows–Wheeler Aligner v0.7.15 (Li and Durbin, 2009). Whole exome sequencing data was retrieved for 2405 CLOZUK2 participants linked to curated pharmacokinetic information.

Genome Analysis Toolkit (GATK) v3.4 (McKenna et al., 2010) was then used for recalibration of base quality scores, realignment around indels and variant calling. Variants were jointly called using GATK HaplotypeCaller and filtered using the GATK variant quality score recalibration tool, VQSR. Processed sequencing data was queried using *Hail* (Hail_Team), and standard quality control procedures were performed, excluding samples where one or more of the following conditions was true: a) less than 70 % of the exome target achieved 10× coverage; b) mismatch between genetically inferred sex and recorded sex; c) presence of another sample with a second degree or closer genetic relationship; d) failed sequencing quality control hard filter. Further details about the sequencing procedure and quality control are presented in the Supplementary Information. Across all sample QC measures (exome coverage and quality, sex-check, ancestry, relatedness, and hard filters), we excluded 343 cases, and 2062 individuals were kept in the final sample and taken forward in our analysis.

### Variant and gene set selection

Variants were annotated using the Ensembl Variant Effect Predictor v102 (McLaren et al., 2016) and CADD v1.6 (Rentzsch et al., 2021) in *Hail*. We selected three classes of functionally important variants for our analyses: synonymous variants, protein truncating variants, and missense variants. Variants annotated as stop-gain, frameshift or splice donor/acceptor variants were grouped into the protein truncating variants class (PTVs). In addition, missense variants with CADD PHRED-score ≥ 20 - a threshold optimised for pharmacogenes (Zhou et al., 2019) - were considered putatively damaging missense variants and included in the missense variant analyses. Variants included in each of those classes (i.e., synonymous, PTVs or damaging missense) with MAF ≤1 % in both the filtered CLOZUK2 sample and the European subset of gnomAD v2.1.1 controls (‘controls_nfe’) (Karczewski et al., 2020) were retained for further analyses. Synonymous variants were expected to have neutral effects, whereas the protein truncating variants and missense classes enrich for variants with putatively deleterious impacts on protein function/activity (Ingelman-Sundberg et al., 2018).

Next, we grouped the variants fulfilling the inclusion criteria into genes and subsequently into pathways or gene sets of interest. As only a relatively small number of genes have evidence of direct roles in clozapine metabolism (Pardiñas et al., 2021), we assessed gene sets relevant to ADME processes and related to clozapine pharmacokinetics (Sim et al., 2011). Four gene sets were selected *a priori* for analysis: the PharmGKB clozapine pathway set, including genes with consensus evidence of involvement in clozapine pharmacokinetics (15 genes); the PharmaADME core set, including genes related to general drug metabolism (33 genes); the PharmaADME extended set, incorporating drug targets (295 genes); and the CPIC Very Important Pharmacogenes (VIP) set, a curated list of genes used to derive pharmacogenomic recommendations for many medications (57 genes). Supplementary Table 1 details the genes included in each of the gene sets. Note that as genes in these sets are involved in similar drug metabolism routes, there is a large degree of overlap, and their union represents 321 unique genes (Supplementary Fig. 3). The frequency of variants fulfilling the inclusion criteria and present in genes included in the pathways or gene sets of interest (Supplementary Table 1) is given in Supplementary Fig. 4.

### Statistical analysis

All statistical analyses were conducted in R v4.2.2. Rare variants located in genes included in the sets listed above were counted and aggregated in a burden metric within each of the four gene sets. This strategy is considered effective for jointly testing multiple rare variants, improving statistical power for association discovery. We then performed burden analysis to assess the relationships between set-based rare variant burdens and plasma concentrations of clozapine (active parent compound) and norclozapine (main metabolite) using generalised linear mixed-effect model (GLMM) regressions. Models were fitted using the *glmmTMB* package (Brooks et al., 2017) in R, which allows for defining fixed-effect predictors or covariates for both the outcome mean and variance. The covariates included for the mean were: clozapine daily dose, the time between the last clozapine dose and blood sample collection (TDS), sex, age, age^2^, 10 genomic principal components derived from the exome sequencing data, 4 index SNPs previously identified in a clozapine metabolite GWAS of overlapping CLOZUK2 samples - rs1126545_T, rs2472297_T, rs61750900_T, rs2011425_G (Pardiñas et al., 2019), and the burden of synonymous variants across the exome at the same MAF threshold. Covariates for the variance included: clozapine dose, TDS, sex, age, and age^2^. One random effect predictor per study participant was included to account for the longitudinal nature of the data and prevent confounding due to non-independence of the plasma concentrations within individuals. Further details about the statistical modelling procedure are given in the Supplementary Information.

Given the high overlap in the genes included in the four gene sets tested, as well as in the variant categories (PTVs, missense or both – PTVs + missense), the Benjamini-Hochberg false discovery rate (FDR) method was used as a correction for multiple testing at a p_FDR_ ≤ 0.1 threshold (van den Oord, 2008). In each regression model, we estimated the variance explained by the rare variant burden using the semi-partial R^2^ method implemented in the *partR2* R package (Stoffel et al., 2021). As this method is not implemented for the generalised *glmmTMB* models described above, standard LMMs were fitted using the *lme4* framework with the same fixed- and random-effect predictors used for this procedure.

We also aimed to assess whether differences in metabolism observed in individuals carrying rare damaging variants in those gene sets would affect their probability of having clozapine plasma concentrations below (<350 ng/mL), within (350–600 ng/mL), or above the therapeutic range (>600 ng/mL). Regression models for these classes of clozapine plasma concentrations were fitted with ordinal GLMMs assuming a cumulative link function for the bins of clozapine plasma concentrations via the *ordinal* R package (Christensen, 2023). This regression included the same fixed-effect and random-effect covariates from the models described above.

Lastly, since the set-based rare variant burden was calculated across several genes, we also performed gene-based analyses using the same method and covariates as the primary analyses in order to disentangle gene-specific effects from the overall set-based associations. In this step, individual genes carrying fewer than 5 damaging variants were not analysed due to lack of power and increased likelihood of false positives. Multiple testing was accounted for using a Bonferroni correction corresponding to the number of genes being evaluated.

## 3 Results

After phenotypic and genetic quality control, 2062 individuals linked to 6585 clozapine plasma concentration assays were retained: 72.8 % were male (1501 male; 561 female), and the mean age was 43.61 years (SD = 11.38) at the last available clozapine monitoring measure. Over 6000 rare damaging nonsynonymous alleles (MAF ≤ 1 %) affecting 313 of 321 genes included in the four gene sets were identified (624 PTVs; 5435 missense SNVs), details about the frequency of rare variants included in our analyses are shown in Supplementary Fig. 4.

### Gene set association analyses

**Fig. 1:**
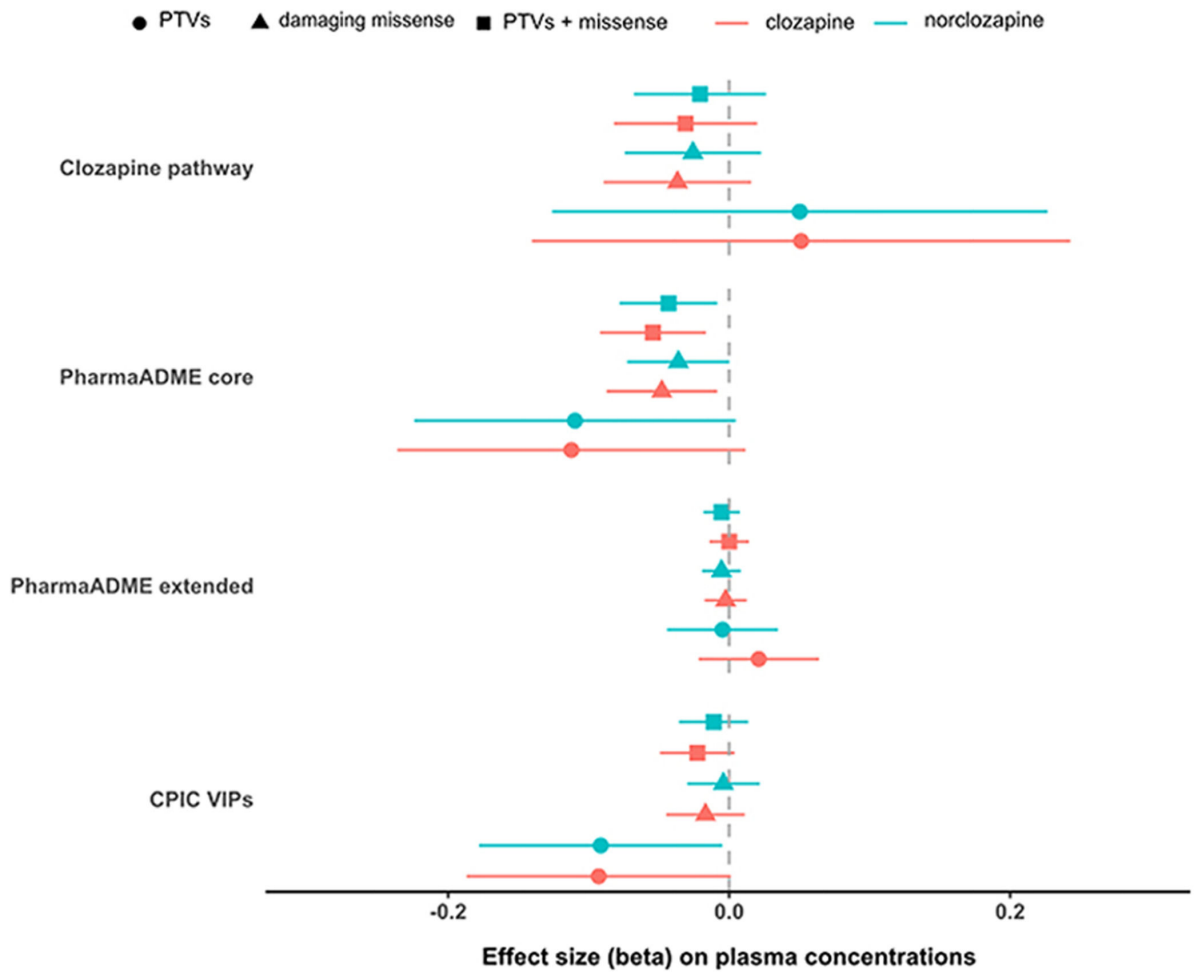
Estimated effect sizes and confidence intervals for the different variant classes in each of the four gene sets analysed. Supplementary Table 2 gives the full results for all gene sets for both clozapine and norclozapine levels, including the percentage of variability explained by rare variants in each of the sets.

We observed significant associations between the burden of rare damaging mutations in the PharmaADME core set and both clozapine and norclozapine plasma concentrations (Fig. 1). The combined PTVs + missense variant set included 837 rare damaging alleles affecting 29 genes in the core PharmaADME set. Each rare allele in this gene set was associated with lower clozapine (β = -0.054, SE = 0.019, *P*-value 0.005, *R*^2^ = 0.407 %) and norclozapine levels (β =−0.043, SE = 0.018, *P*-value = 0.015, *R*^2^ = 0.332 %) when compared to individuals carrying zero damaging alleles, though only the clozapine association remained significant after FDR correction. Comparing these coefficient estimates with the daily dose predictor in the same statistical model (Supplementary Table 3), we estimate that the impact of a single damaging allele in this gene set on clozapine plasma concentrations is akin to a reduction in clozapine dose of about 36 mg/day (Supplementary Fig. 5). Supplementary Table 2 details the main results for all gene sets for both clozapine and norclozapine levels, including the percentage of pharmacokinetic variance explained by rare variants in these sets.

**Fig. 2:**
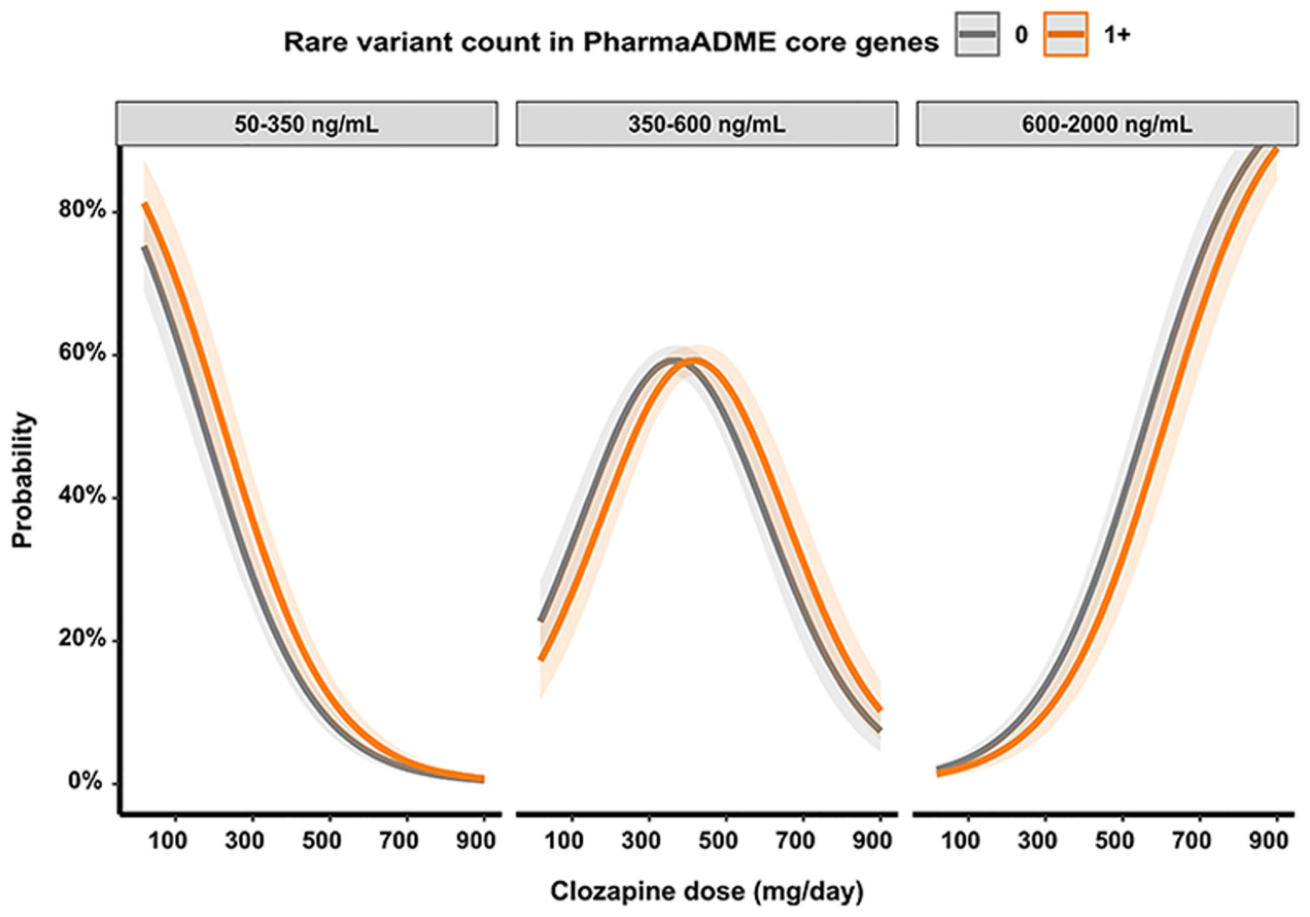
Marginal effects derived from an ordinal mixed model regression for the probability of achieving clozapine concentrations in or out of the therapeutic range for individuals carrying none or at least one rare damaging variant in the PharmaADME core gene list. Shaded areas indicate a 95 % confidence interval. Supplementary Fig. 5 details effects for individuals carrying different numbers of rare damaging variants in the PharmaADME core gene set in the relationship between clozapine doses and plasma concentrations.

We found that individuals carrying at least one rare damaging variant in PharmaADME core genes were 30 % less likely than non-carriers to reach clozapine concentrations within or above the therapeutic range throughout their monitoring (OR = 0.694; SE = 0.145; *P* = 0.012). Fig. 2 shows that those carrying rare damaging alleles were at increased likelihood of presenting subtherapeutic clozapine concentrations, requiring doses of 275 mg/day to reach the therapeutic interval with at least 50 % probability (50.43 %; SE 2.48 %). Individuals without any damaging rare variant had similar probabilities of achieving therapeutic concentrations at 225 mg/day (50.44 %; SE 2.06 %).

### Gene-based association analyses

Next, we conducted a follow-up gene-based burden analysis focused on the genes within the PharmaADME core set (see Supplementary Table 1). In total, 26 genes were included in this step, and 3 were excluded due to having fewer than 5 rare damaging nonsynonymous variants. No single gene was significantly associated with clozapine or norclozapine plasma concentrations after multiple testing corrections (α = 0.05/26 - Supplementary Tables 4 and 5). We observed that rare damaging variants in *CYP1A2* displayed the strongest nominally significant associations with higher clozapine (β = 0.324, SE = 0.124, *P* = 0.009, *R*^2^ = 0.233 %) and norclozapine (β = 0.320, SE = 0.115, *P* = 0.005, *R*^2^ = 0.235 %) concentrations. Other nominal gene-level associations observed were *UGT1A1* with clozapine levels (β = −-0.258, SE = 0.122, *P* = 0.034, R^2^ = 0.144 %) and *SULT1A1* with norclozapine (β = 0.281, SE = 0.137, *P* = 0.041, R^2^ = 0.036 %).

**Fig. 3:**
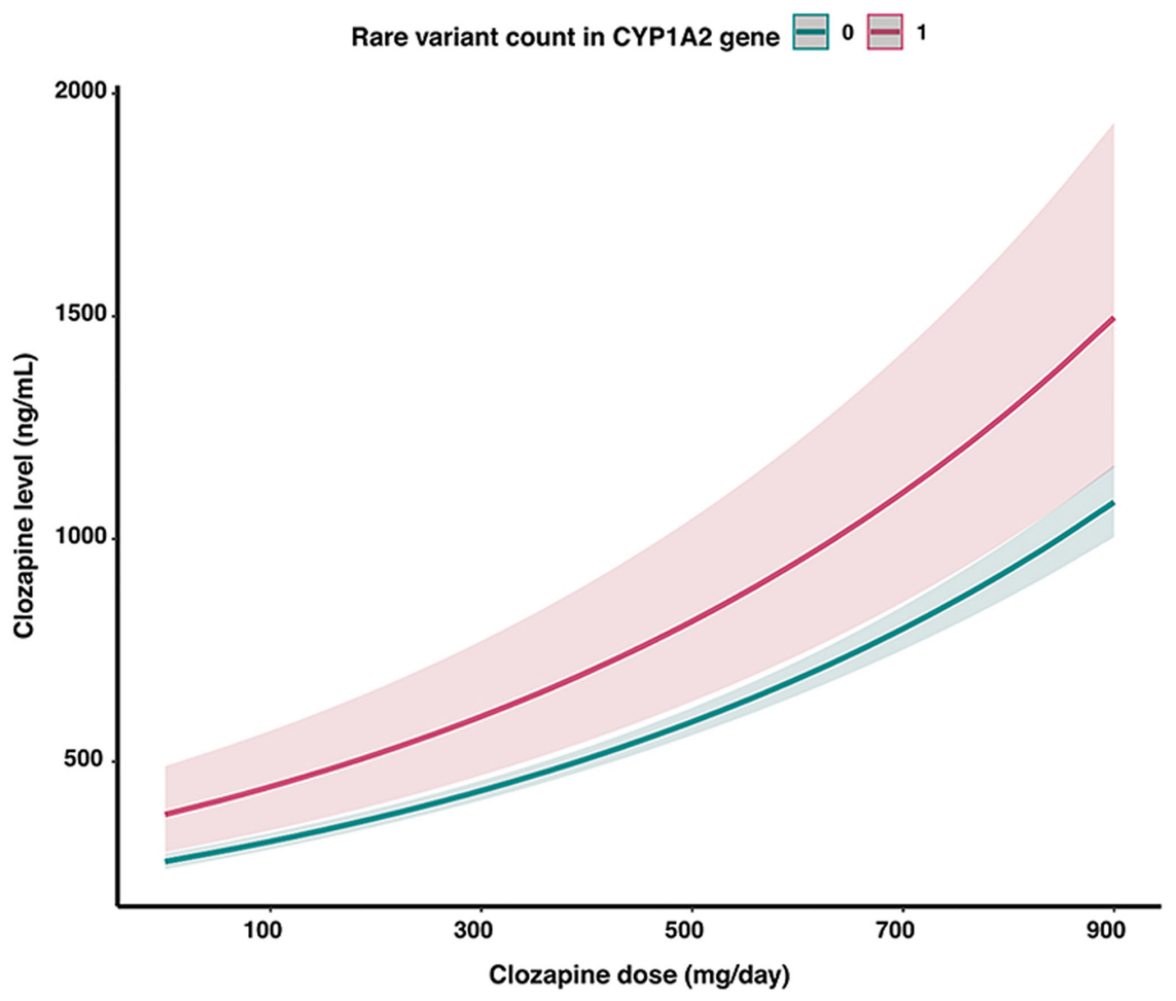
Marginal effects representing the relationship between clozapine doses and plasma concentrations in individuals carrying or not rare damaging variants in the *CYP1A2* gene. Shaded areas highlight a 95 % confidence interval for the estimates.

Rare alleles in *CYP1A2* variants had an opposite direction of effect to rare alleles in the full PharmaADME core set (Fig. 3 – Supplementary Table 6), that is, they were associated with higher clozapine and norclozapine levels. For this reason, we performed a sensitivity analysis by excluding *CYP1A2* from the PharmaADME set, recalculating the burden scores, and conducting the GLMM regression again. This yielded the same result of rare variant burden in the PharmaADME core set being associated with clozapine and norclozapine metabolism, and as expected, the effect sizes were slightly stronger after the removal of *CYP1A2* (Supplementary Fig. 6). We also tested for additive effects by separately considering rare damaging variants in *CYP1A2* and the remainder of the PharmaADME gene set, showing that adding both burdens as individual predictors resulted in an 80.83 % increase in the variance of clozapine levels explained by rare genetic variation (*CYP1A2* + PharmaADME core gene set R^2^ = 0.736 %). We did not find any evidence of multiplicative (i.e., non-linear) relationships between these two predictors in our dataset (interaction test *P* = 0.470).

## 4 Discussion

To our knowledge, this is the largest sequencing study seeking to evaluate the effects of rare pharmacogenomic variants in clozapine metabolism. Using longitudinal clozapine monitoring data, we evaluated WES data to discover genes and gene sets harbouring rare variants associated with inter-individual variability in drug metabolism. We provide evidence that the burden of deleterious rare variants in pharmacogenes can significantly affect clozapine metabolism, leading to different metabolite plasma concentrations for individuals prescribed the same drug dose.

In this retrospective analysis of data from a real-world clinical setting, we identified that a greater burden of rare damaging variants in the PharmaADME core gene set, which contained 30 genes involved in general drug metabolism (Supplementary Table 1), was associated with significantly lower clozapine and norclozapine plasma concentrations (Fig. 1). We estimate that the burden of rare variants in this set explains 0.40 % of the variability of this phenotype, a similar figure to the 0.61 % variance explained by a PRS generated from genome-wide significant loci in a recent clozapine metabolism GWAS performed in this sample (Pardiñas et al., 2023). The variability in clozapine plasma levels explained by the presence of these rare variants is small, which is expected since this metric is dependent on the frequency of such alleles in the population, however, they follow the pattern expected for other pharmacogenomic predictors of drug concentrations (Nelson et al., 2016). Despite this, at an individual level, the effect of carrying rare damaging alleles in this set on clozapine levels is comparable in magnitude to other known clinical and demographic variables, with the effect sizes being similar to a 36 mg reduction in clozapine daily dose (Supplementary Table 3). As our models assume a linear additive effect for the mutation burden, carriers of multiple rare alleles (the maximum seen in our study was 3) would require further dose adjustments to match the levels expected in non-carriers (Supplementary Fig. 5). A context for this result is the observation that metabolic networks have properties that make them resistant to deactivation by deleterious genetic variation (Khurana et al., 2013), which in turn makes them more likely to tolerate protein-damaging variants. Our findings suggest that individuals accumulating several rare damaging variants (as assessed in the gene set burden scores) might have impaired biochemical functions relevant to drug response or even downstream disease risk (Bomba et al., 2022). Similarly, other studies have shown that the burden of rare variants in pharmacogenes could be a predictor of pharmacological treatment outcomes (De Mattia et al., 2023; De Mattia et al., 2022; Gray et al., 2022; Xiao et al., 2020).

Furthermore, through ordinal mixed-model regression analysis, we also observed that carriers of at least one damaging allele affecting genes in the PharmaADME core set (*N* = 627) were 30 % less likely than non-carriers to reach the therapeutic range (350–600 ng/mL) of plasma concentrations of clozapine (OR = 0.694; SE = 0.145; *P* = 0.012; Fig. 2). The clinical management of individuals being treated with clozapine often focuses on maintaining clozapine plasma concentrations within the standard therapeutic range (Flanagan et al., 2023a; Siskind et al., 2021); some guidelines suggest that this can be better achieved by stratifying dosing according to known predictors of clozapine metabolism (Correll et al., 2022; de Leon, 2022; de Leon et al., 2022). Using this approach, we observed that by stratifying individuals according to the presence of rare variants, individuals not carrying any damaging rare variant had a 50 % probability of achieving clozapine concentrations within the therapeutic range at a dose of 225 mg/day, while in carriers of damaging rare alleles in the PhamaADME genes, the corresponding dosage was 275 mg/day.

We further analysed the burden of rare variants within specific genes included in the PharmaADME core gene set. Although no single gene was significantly associated with clozapine or norclozapine plasma levels after Bonferroni correction for multiple testing, the most significant gene in this analysis was *CYP1A2* (*P* = 0.009). However, individuals carrying rare variants in *CYP1A2* showed higher plasma clozapine levels when taking the same clozapine dose as non-carriers (Fig. 3). This was contrary to the PharmaADME core gene set results and prompted us to test for individual contributions to clozapine metabolism of rare variants within and outside *CYP1A2* (Supplementary Fig. 6). *CYP1A2* encodes the main catalyst enzyme of the clozapine to norclozapine biotransformation, and while common variants in its proximity have been previously associated with clozapine metabolite levels (Pardiñas et al., 2019; Smith et al., 2020), no such association yet exists for coding SNVs.

Although genetic factors that account for a significant proportion of the inter-individual variability in antipsychotic drug metabolism have been suggested as useful to improve therapeutic response and avoid ADRs (Yoshida and Müller, 2019), an effective model for the clinical implementation of this information in guiding treatment choices is not yet available, and recent revisions of clozapine treatment guidelines do not currently consider genetic variants relevant for clinical management (Correll et al., 2022; de Leon, 2022; de Leon et al., 2022). Our results suggest convergence of rare and common variants in genes involved in pharmacokinetic pathways affecting clozapine first-pass metabolism and implicate novel variants in generating atypical metaboliser status. In doing so, it supports the view that, in the future, a precision psychiatry framework might benefit from taking into account (pharmaco)genetic variation beyond the traditional PGx star allele definitions assessed by candidate gene studies and commercial genetic testing companies (Ingelman-Sundberg et al., 2018; Pardiñas et al., 2019; Zhou and Lauschke, 2022). While our study is not powered to assess the strength of specific rare variants, and external replication and validation is needed to understand how these genetic markers are associated to drug metabolism, the effects we detect for the mutational burdens are of a sufficient magnitude to be relevant for particular subsets of the population of those on clozapine, such as those who only tolerate low drug doses or people initiating treatment, as serious adverse effects are more common in the initial phases of clozapine treatment and could be potentially ameliorated by a personalised dose titration scheme (Flanagan et al., 2020). This knowledge, added to that coming from common variant analyses, might be incorporated into clozapine prescribing guidelines that at present rely on non-genetic characteristics to personalise dosing (de Leon et al., 2022). In the meantime, proactive therapeutic drug monitoring strategies remain a tried-and-tested approach for navigating the large inter-individual variability of clozapine metabolism and achieving beneficial clinical outcomes (Flanagan et al., 2023a; Molden, 2021).

One limitation of this study is that rare variant analyses require a large sample size to allow identifying of single rare variants that are associated with complex traits. Although we had access to WES data, we chose to focus our efforts on a relatively small number of genes with robust *a priori* evidence of their involvement in drug metabolism, attempting to avoid limiting the power of our statistical testing through multiple testing. This strategy followed the rationale and results from other genomic studies of drug metabolism, showing that these traits are likely influenced by a small number of variants in relevant genes, each presenting with a relatively large effect (Pardiñas et al., 2019; Timpson et al., 2018). However, unlike candidate studies, which often have included variants with experimentally derived functional activity information, we selected variants based on in-silico functional approaches such as protein impact prediction algorithms, and thus most of them have not yet been functionally assessed (Supplementary Table 7). This is a PGx-informed approach to variant prioritisation, selecting rare variants putatively affecting enzyme and transporter function (Bush et al., 2016; Zhou et al., 2019), but can still include markers with little or no impact on gene product while omitting other potentially important variants. Thus, assessing and collecting more information on the functional genetic makeup of pharmacogenes, through either bioinformatics or direct experimental approaches (Brunk et al., 2018; Kim et al., 2022), will be valuable to update these analyses and for further research into developing PGx biomarkers for metabolite pharmacokinetics and drug response.

Another limitation is that, the regression models fitted for gene- and set-based analyses assume that all rare variants included in the burden scores follow a similar direction of effects (Li and Leal, 2008). This is known not to be the case in disease studies (i.e., both risk-increasing and protective variants can exist) and is likely a simplification for metabolism as well. While, again, our sample size limits the testing we can reliably perform for individual genes, results from gene-based analyses, particularly for *CYP1A2*, suggest that future studies might benefit from statistical methods allowing for testing variants with multiple directions of effect.

Finally, due to the limited phenotypical data available for CLOZUK2, information was not available on a number of important factors known to influence clozapine metabolism, such as cigarette smoking, concomitant use of other medications, and dosing frequency regimen. These factors are associated to inter-individual differences in clozapine metabolism and thus might mediate some of our results. The fact that we could not directly ascertain these predictors is a clear limitation of our study; however, some of the covariates already included in our regression models might capture some of the variance due to these exposures. For example, previous studies have found that sex can be a partial marker of oral contraceptive use and smoking, both interactors of CYP1A2, capturing some of their effects on drug metabolism (Diaz et al., 2005; Ramsey et al., 2016). A related issue is that even though the CLOZUK2 cohort is derived from the diverse current population of the UK, the majority of individuals for which both WES and clozapine levels monitoring data were available were of European ancestry. Given that individuals of other biogeographical ancestries are known to have different clozapine pharmacokinetic profiles (Pardiñas et al., 2023), our study cannot assess a potential rare variant contribution to those known ancestral differences, and its results might not be straightforwardly transferrable to non-European populations. We note, however, that consistency of variant effect sizes across populations is a reasonable assumption for pharmacogenes (Nagar et al., 2020) and that the occurrence of deleterious variation in these gene sets is frequent (though individually rare) across continental ancestries (Scharfe et al., 2017). Thus, the association between rare damaging burden and clozapine metabolism identified here could be relevant beyond the European sample in which it was discovered.

In conclusion, we found that the burden of rare damaging variants within genes in drug metabolism pathways was associated with differences in clozapine metabolite plasma concentrations, explaining part of the genetic variability in clozapine metabolism phenotypes. These differences were comparable to those inferred for other genetic and non-genetic predictors in ours and other studies (Flanagan et al., 2023b; Pardiñas et al., 2023), and despite the rarity of these variants at the population level they could be individually meaningful for their carriers. Our results suggest that pharmacogenomic efforts trying to predict clozapine metabolism and personalise drug therapy could thus benefit from including rare damaging variants in pharmacogenes beyond those already identified and catalogued as PGx star alleles. Further investigation is warranted to confirm and replicate these findings before they can be evaluated in prospective trials or considered in clinical settings.

## Supplementary Material

Supplementary material associated with this article can be found, in the online version, at doi: 10.1016/j.euroneuro.2023.12.007.

Supplementary Material
