# Supplementary material for: Rare variants in pharmacogenes influence clozapine metabolism in individuals with schizophrenia
Source: Eur Neuropsychopharmacol. Author manuscript; Available in PMC 2026 Jun 2. (PMC7619122; doi:10.1016/j.euroneuro.2023.12.007)
Supplement: Supplementary Material [file EMS214093-supplement-Supplementary_Material.docx]

**Rare Variants in Pharmacogenes Influence Clozapine Metabolism in Individuals with Schizophrenia**

Djenifer B. Kappel 1, Elliott Rees 1, Eilidh Fenner 1, Adrian King 2, John Jansen 3, Marinka Helthuis 3, Michael J. Owen 1, Michael C. O’Donovan 1, James T.R. Walters 1, Antonio F. Pardiñas 1*

Affiliations

1. Centre for Neuropsychiatric Genetics and Genomics, Division of Psychological Medicine and Clinical Neurosciences, School of Medicine, Cardiff University, Cardiff, United Kingdom

2. Magna Laboratories Ltd., Ross-on-Wye, United Kingdom

3. Leyden Delta B.V., Nijmegen, the Netherlands

* Corresponding Author

Antonio F. Pardiñas

Email: [PardinasA@cardiff.ac.uk](mailto:PardinasA@cardiff.ac.uk) | Tel: +44 02920 688407

Centre for Neuropsychiatric Genetics and Genomics, Division of Psychological Medicine and Clinical Neurosciences, Hadyn Ellis Building, Maindy Road, Cardiff University, Cardiff, UK, CF24 4HQ

[Supplementary Methods 2](#_Toc144384061)

[Exome capture 2](#_Toc144384062)

[Variant quality control 2](#_Toc144384063)

[Sex check 2](#_Toc144384064)

[Relatedness 3](#_Toc144384065)

[Hard Filters 3](#_Toc144384066)

[Ancestry prediction and principal components estimation 3](#_Toc144384067)

[Variant annotation 4](#_Toc144384068)

[Supplementary Results 4](#_Toc144384069)

[Supplementary Figure 1 6](#_Toc144384070)

[Supplementary Figure 2 7](#_Toc144384071)

[Supplementary Figure 3 8](#_Toc144384072)

[Supplementary Figure 4 9](#_Toc144384073)

[Supplementary Figure 5 10](#_Toc144384074)

[Supplementary Figure 6 11](#_Toc144384075)

[Supplementary Table 1 12](#_Toc144384076)

[Supplementary Table 2 13](#_Toc144384077)

[Supplementary Table 3 14](#_Toc144384078)

[Supplementary Table 4 15](#_Toc144384079)

[Supplementary Table 5 16](#_Toc144384080)

[Supplementary Table 6 17](#_Toc144384081)

[Supplementary Table 7 18](#_Toc144384082)

[Supplementary Table 8 19](#_Toc144384083)

[References 20](#_Toc144384084)

## Supplementary Methods

### Exome capture

Samples were prepared for whole exome sequencing (WES) using the Illumina HiSeq 3000/4000 capture kits according to the manufacturer’s protocol. Once prepared, the exome-captured library was then sequenced in the Illumina HiSeq platform using the paired-end method. Exome sequences had a median of 83% of all targeted bases covered at ≥10×, and samples were excluded if less than 70% of the exome target achieved 10× coverage. Raw data was processed to remove adaptors and low-quality reads, then aligned to the GRCh37 human reference genome with Burrows–Wheeler Aligner (bwa) v0.7.15 (Li and Durbin, 2009). Genome Analysis Toolkit (GATK) v3.4 (McKenna et al., 2010) was then used for recalibration of base quality scores, realignment around indels and variant calling (HaplotypeCaller).

### Variant quality control

We then proceeded with the variant and genotype quality control procedures in Hail (Hail_Team). Initial processing removed variants failing the GATK(McKenna et al., 2010) Variant Quality Score Recalibration (VQSR). The genotypes in the remaining variants were then filtered for depth (DP) ≥ 10, genotype quality score (GQ) ≥ 30, allelic balance (AB) < 0.1 in homozygous calls for the reference allele, AB ≥ 0.25 and ≤ 0.75 for heterozygous calls and AB ≥ 0.9 for homozygous calls for the alternative allele. Variants were also excluded if their call rate was < 0.97 or had a Hardy–Weinberg Equilibrium exact test P value of < 1 × 10^−6^.

### Sex check

Genetic sex was inferred using a set of high-quality common variants on the X and Y chromosomes and from the rates of heterozygous and homozygous calls on the X chromosome using *Peddy(Pedersen and Quinlan, 2017)*. A sex check procedure compared genetically imputed sex with recorded sex on phenotype information. A total of 15 individuals were excluded due to their inferred sex not matching their recorded sex.

### Relatedness

The PC-Relate method (Conomos et al., 2016), implemented in *Hail*, was used to assess genetic relatedness between all samples. First, pairwise kinship coefficients were estimated between all pairs of samples using LD-pruned SNPs (max r^2^ < 0.5) from a set of high-quality common variants (MAF > 5%). Next, the pairwise kinship coefficient was used to identify related individuals with a second-degree or closer relationship. Once such a relationship was inferred, the sample with a higher sequencing quality and more phenotypical information from each pair was retained for further analyses. This procedure eliminated 89 samples.

### Hard Filters

Overall sample quality control was assessed using *Hail’s* sample_qc function to generate sample metrics from the raw variant calls. Several metrics were assessed, and hard-call filters were derived to exclude low-quality samples and individual outliers. Samples were required to have call rates above 0.9, and individuals above or below 3 SD from the sample mean for each of the following were removed: number of SNPs (nSNPs), heterozygous-homozygous call ratio (rHetHomVar), number of singleton calls (nSingleton), transition-transversion ratio (rTiTv), and insertion-deletion (rInsertionDeletion). The hard filter QC removed 75 individuals who were outliers for one or more of the above metrics (**Supplementary Figure 1**).

### Ancestry prediction and principal components estimation

﻿ *Peddy* was also used to infer the biogeographical ancestry of CLOZUK2 samples using Principal Components Analysis (PCA) and a support vector machine guided by samples of known ancestry from the thousand genomes project (1KG) (Genomes Project et al., 2015). The majority of individuals (99.5%) were classified by the algorithm as European, with only three samples classified as American and 9 as Admixed/Other. In order to avoid population stratification, a potential problem in rare variant studies, we excluded samples falling 4 SD out of the mean of PC1 and PC2 for European samples (**Supplementary Figure 2**). This procedure removed 10 individuals, of which 9 had been classified as European.

We then used the same subset of high-quality common variants (MAF > 5%) used to estimate relatedness to calculate 10 genetic principal components using *Hail’s* Hardy-Weinberg-normalized PCA method (hl.hwe_normalized_pca). These PCs were later used as covariates in all regression analysis.

### Variant annotation

After extensive quality control, variants were annotated using the Ensembl Variant Effect Predictor v102^14^ and CADD v1.6 (Rentzsch et al., 2021; Rentzsch et al., 2019) in *Hail*. Variants annotated as stop-gain, frameshift or splice donor/acceptor variants were grouped into the protein truncating variants class (PTVs). In addition, missense variants with CADD PHRED-score ≥ 20 were considered putatively damaging missense variants and included in the missense variant analyses. Variants included in each of those classes (i.e., PTVs or damaging missense) and presenting at a minor allele frequency (MAF) lower than 1% in both the filtered CLOZUK2 sample and the European subset of gnomAD v2.1.1 (Karczewski et al., 2020) controls (‘controls_nfe’) were retained for further analyses. The frequency of variants fulfilling the above criteria and present in genes included in the pathways or gene sets of interest (**Supplementary Table 1; Supplementary Figure 3**) is given in **Supplementary Figure 4.**

## Supplementary Results

The implementation of GLMMs in *glmmTMB*(Brooks et al., 2017) allows for, in addition to standard mixed-effect models with fixed and random effect terms, fitting a wider class of “distributional regression” models (Kneib et al., 2021). These flexible models can be used to estimate the effects of predictors for the conditional mean of the outcome (sometimes referred to as “location”) and/or its variance (“dispersion” or “scale”). This can increase the power of regression approaches by reducing the overall unaccounted residual (“error”) variance, part of which can otherwise be captured by random effect terms (de Villemereuil et al., 2018) in GLMMs, as well as increasing the precision of estimates derived from fixed-effect terms (Walters et al., 2018). Taking advantage of this feature of *glmmTMB*, we included predictors previously shown to influence the within-person variance of clozapine plasma concentrations (Diaz et al., 2005; Jakobsen et al., 2017) in all our regression models: Clozapine dose, the time between the last clozapine dose and blood sample collection (TDS), sex, age, and age². **Supplementary** **Table 3** shows the results for the PharmaADME core full regression model, with the estimated effect sizes and corresponding summary statistics for predictors of the mean and variance of clozapine levels. The inclusion of these predictors improved the fit of our models evidenced by a reduction in the RMSE (Root Mean Squared Error) statistic from 164.3 in the standard mixed-effect models to 163.8 in the model including both mean and variance parameters.

Further to our main analyses, we tested if the presence of rare damaging variants in the PharmaADME core genes could simultaneously impact the mean and variance of clozapine plasma concentrations, as carriers of these variants might have a larger within-subject variability due to their atypical metabolism. We indeed found evidence of this, but only for those carrying PTVs in the PharmaADME core list (**Supplementary Table 8**). However, these results should be evaluated cautiously as testing for variance predictors in a mixed-effect model setting is likely less powered than testing for predictors of the mean (Roberson et al., 2007; Walters et al., 2018), and only a small subset of individuals carried those variants in our sample (n= 73). With this consideration, PTV carriers being more likely to have larger variability in clozapine levels further suggests that the presence of these variants impacts clozapine metabolism and supports the utility of therapeutic drug monitoring (TDM) approaches for personalising clozapine prescriptions while rare genetic variation becomes a part of pre-emptive pharmacogenomic testing.


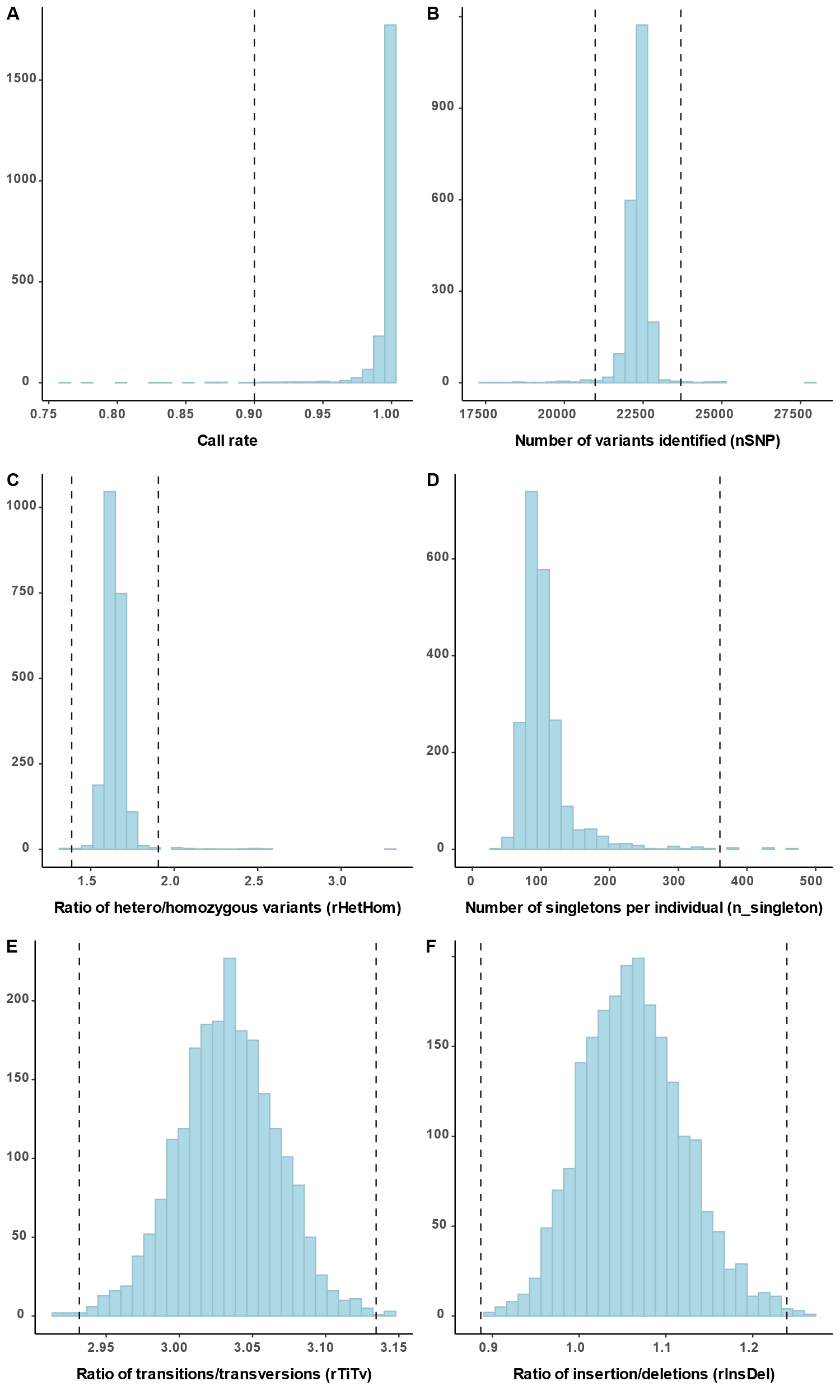


**Supplementary Figure 1**: Histograms showing the distributions of sample metrics used in the hard filter QC. The dashed lines indicate the thresholds used to filter samples from the analysis.

**
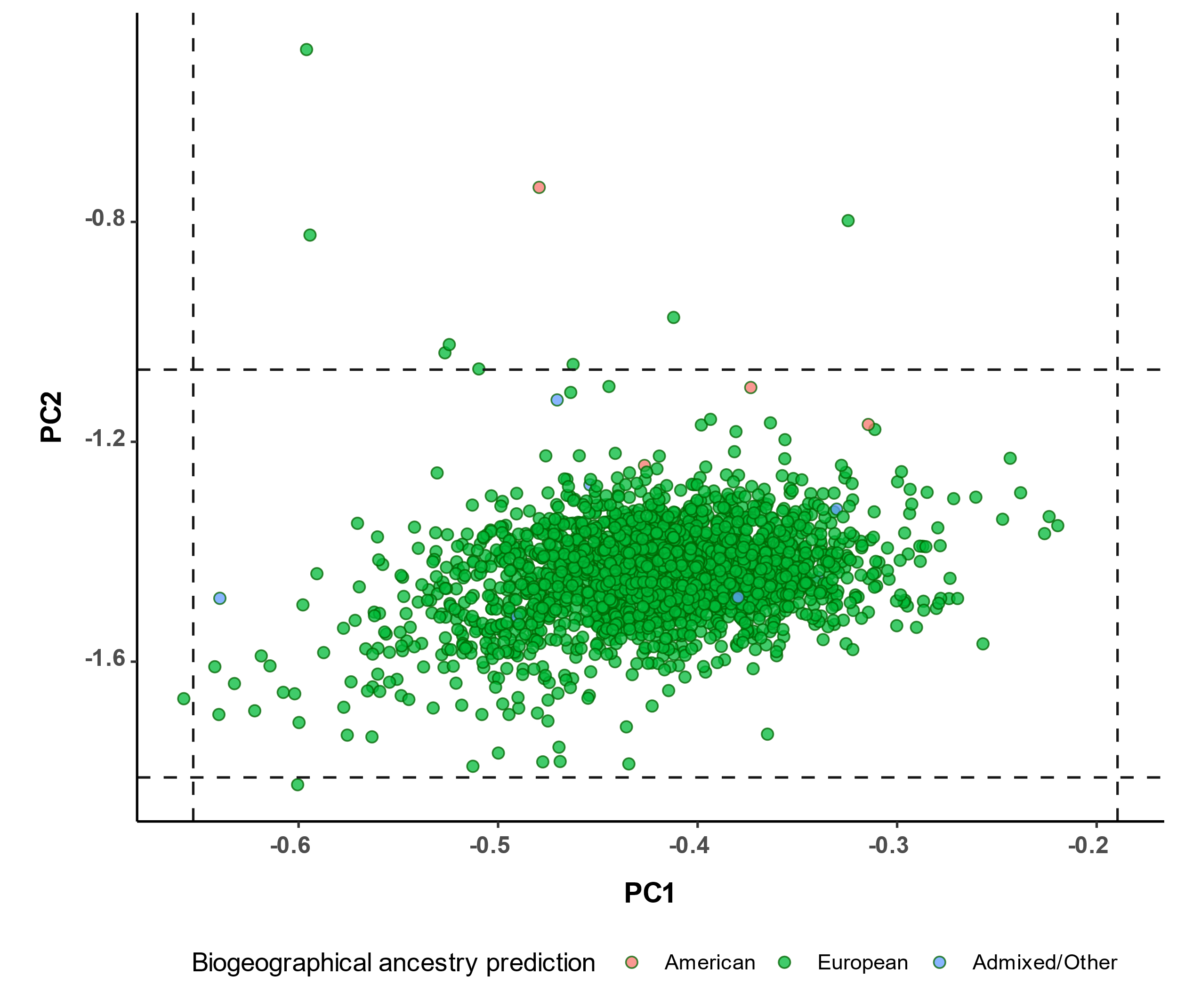
**

**Supplementary Figure 2**: Principal components analysis of CLOZUK2 samples. The dashed lines show the thresholds used to exclude samples to avoid population stratification. Colours represent the individual’s biogeographical ancestry prediction derived in *Peddy*.


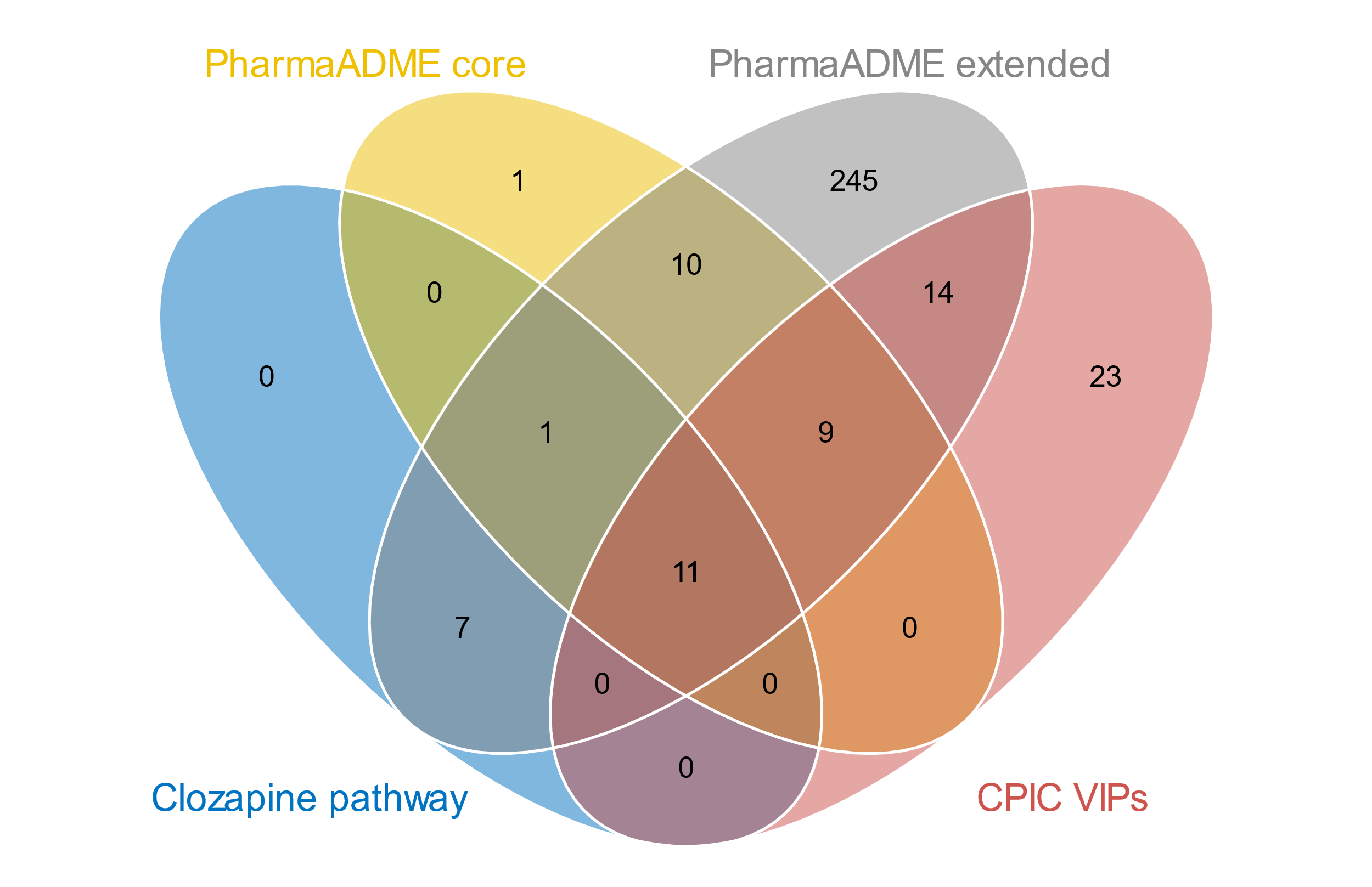


**Supplementary Figure 3**: Venn diagram showing the intersection of all gene sets analysed.


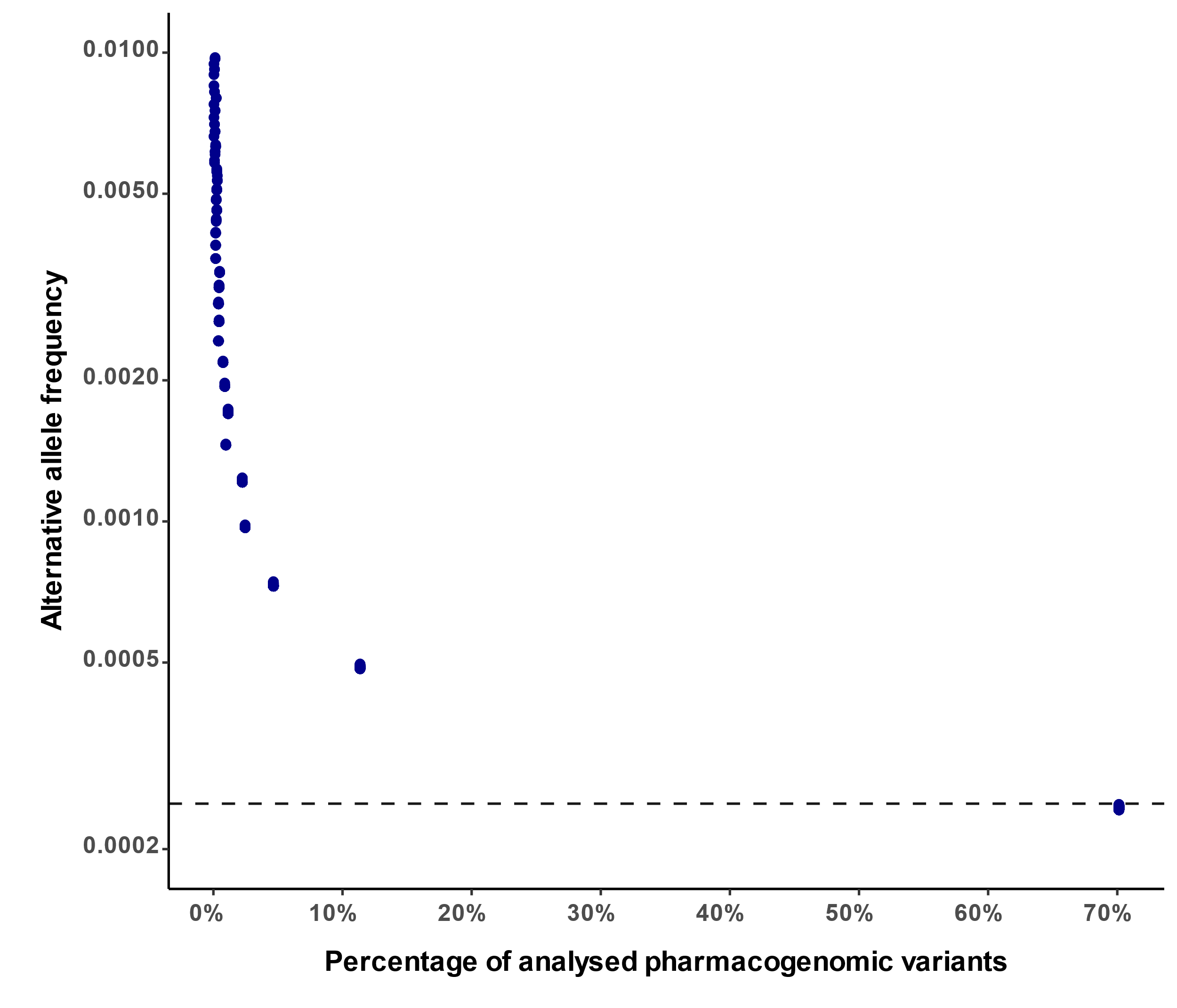


**Supplementary Figure 4**: The frequency of variants fulfilling all inclusion criteria (MAF <1%; likely damaging; overlapping genes in the predefined sets) is depicted above. The dashed line represents the alternative allele frequency of singletons in this specific dataset. Approximately 70% of the variants included in our analyses were only found in a single individual.


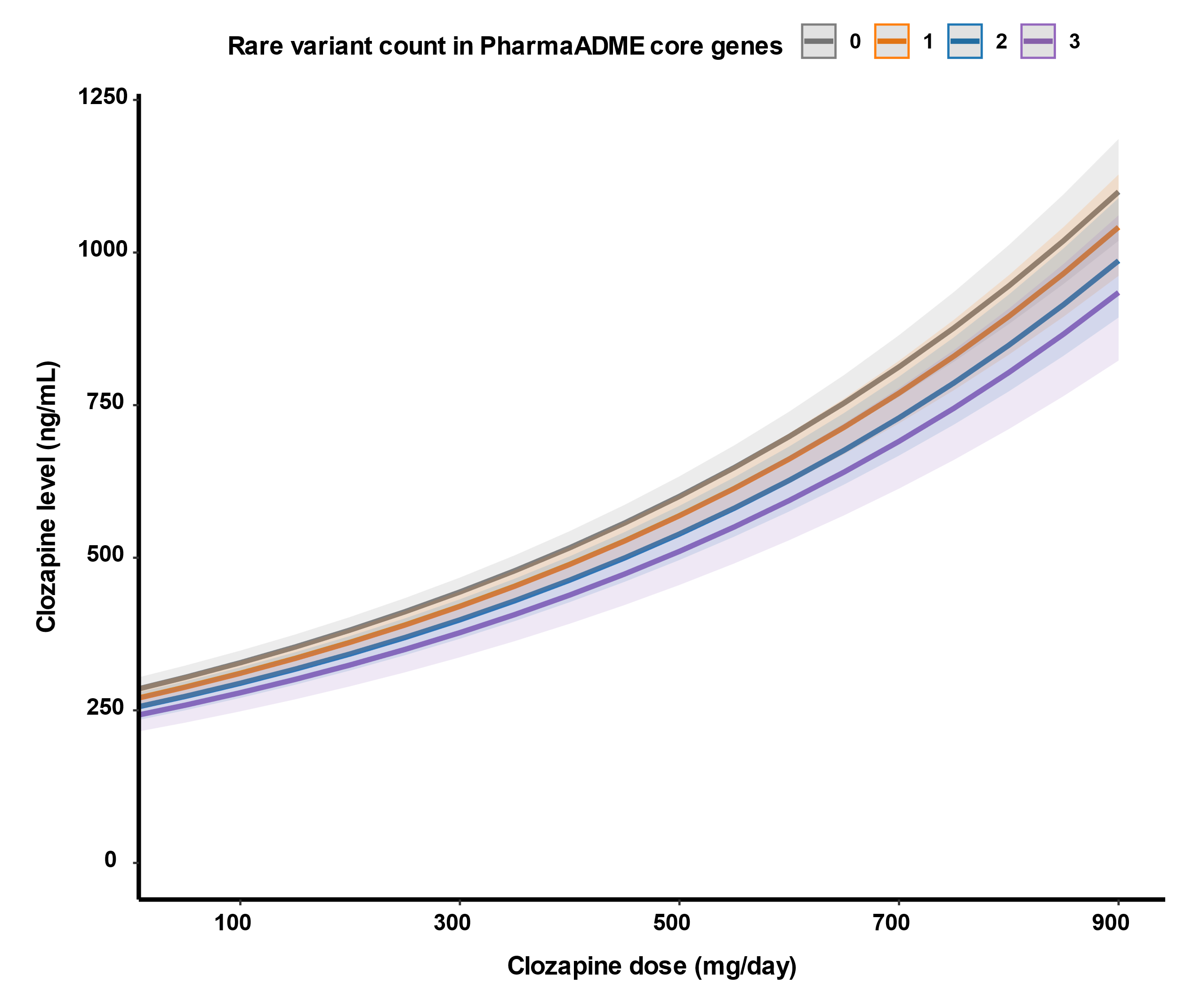


**Supplementary Figure 5**: Marginal effects for individuals carrying different numbers of rare damaging variants in the PharmaADME core gene set in the relationship between clozapine doses and plasma concentrations. Lines indicate the estimated regression effect size and the shaded area its corresponding 95% confidence interval at specific doses.


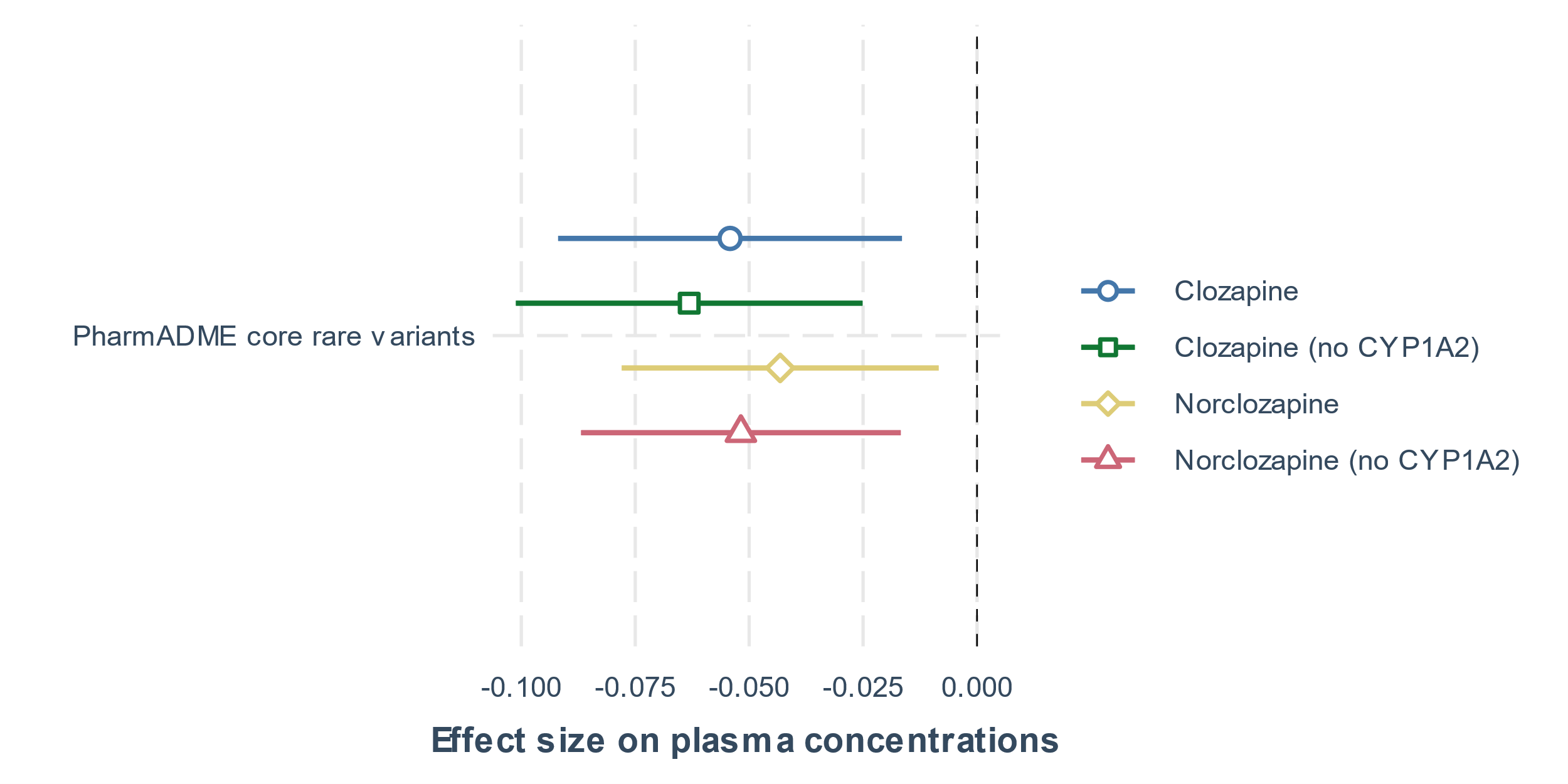


**Supplementary Figure 6**: Differences in the effect size of rare damaging variants in PharmaADME core set list on clozapine metabolism phenotypes when including or removing *CYP1A2* variants.

**Supplementary Table 1**: Genes included in the pharmacogenomic gene sets analysed

| **Gene sets** | **Genes included** |
| --- | --- |
| Clozapine pathway | *ACB1, ABCC1, ABCG2, CYP1A2, CYP2C19, CYP2C9, CYP2D6, CYP2E1, CYP3A4, CYP3A43, CYP3A5, FMO3, SLC22A1, SLC22A2, SLC22A3, UGT1A1, UGT1A3, UGT1A4, UGT2B10* |
| PharmaADME core | *ABCB1, ABCC2, ABCG2, CYP1A1, CYP1A2, CYP2A6, CYP2B6, CYP2C19, CYP2C8, CYP2C9, CYP2D6, CYP2E1, CYP3A4, CYP3A5, DPYD, GSTM1, GSTP1, GSTT1, SLC6A2, NAT2, SLC15A2, SLC22A1, SLC22A2, SLC22A6, SLCO1B1, SLCO1B3, SULT1A1, TPMT, UGT1A1, UGT2B15, UGT2B17, UGT2B7* |
| PharmaADME extended | *ABCA1, ABCA4, ABCB1, ABCB11, ABCB4, ABCB5, ABCB6, ABCB7, ABCB8, ABCC1, ABCC10, ABCC11, ABCC12, ABCC13, ABCC2, ABCC3, ABCC4, ABCC5, ABCC6, ABCC8, ABCC9, ABCG1, ABCG2, ADH1A, ADH1B, ADH1C, ADH4, ADH5, ADH6, ADH7, ADHFE1, AHR, ALDH1A1, ALDH1A2, ALDH1A3, ALDH1B1, ALDH2, ALDH3A1, ALDH3A2, ALDH3B1, ALDH3B2, ALDH4A1, ALDH5A1, ALDH6A1, ALDH7A1, ALDH8A1, ALDH9A1, AOX1, ARNT, ARSA, ATP7A, ATP7B, CAT, CBR1, CBR3, CDA, CES1, CES2, CFTR, CHST1, CHST10, CHST11, CHST12, CHST13, CHST2, CHST3, CHST4, CHST5, CHST6, CHST7, CHST8, CHST9, CYB5R3, CYP11A1, CYP11B1, CYP11B2, CYP17A1, CYP19A1, CYP1A1, CYP1A2, CYP1B1, CYP20A1, CYP21A2, CYP24A1, CYP26A1, CYP26C1, CYP27A1, CYP27B1, CYP2A13, CYP2A6, CYP2A7, CYP2B6, CYP2C18, CYP2C19, CYP2C8, CYP2C9, CYP2D6, CYP2D7, CYP2E1, CYP2F1, CYP2J2, CYP2R1, CYP2S1, CYP39A1, CYP3A4, CYP3A43, CYP3A5, CYP3A7, CYP46A1, CYP4A11, CYP4B1, CYP4F11, CYP4F12, CYP4F2, CYP4F3, CYP4F8, CYP4Z1, CYP51A1, CYP7A1, CYP7B1, CYP8B1, DDO, DHRS1, DHRS12, DHRS13, DHRS2, DHRS3, DHRS4, DHRS4L1, DHRS4L2, DHRS7, DHRS7B, DHRS7C, DHRS9, DHRSX, DPEP1, DPYD, EPHX1, EPHX2, FMO1, FMO2, FMO3, FMO4, FMO5, FMO6P, GPX1, GPX2, GPX3, GPX4, GPX5, GPX6, GPX7, GSR, GSS, GSTA1, GSTA2, GSTA3, GSTA4, GSTA5, GSTCD, GSTK1, GSTM1, GSTM2, GSTM3, GSTM4, GSTM5, GSTO1, GSTO2, GSTP1, GSTT1, GSTT2, GSTZ1, HAGH, HNF4A, HNMT, HSD11B1, HSD17B11, HSD17B14, IAPP, KCNJ11, MAT1A, METAP1, MGST1, MGST2, MGST3, MPO, NAT1, NAT2, NNMT, NOS1, NOS2, NOS3, NR1I2, NR1I3, PDE3A, PDE3B, PLGLB1, PNMT, PON1, PON2, PON3, POR, PPARA, PPARD, PPARG, RXRA, SERPINA7, SLC10A1, SLC10A2, SLC13A1, SLC13A2, SLC13A3, SLC15A1, SLC15A2, SLC16A1, SLC19A1, SLC22A1, SLC22A10, SLC22A11, SLC22A12, SLC22A13, SLC22A14, SLC22A15, SLC22A16, SLC22A17, SLC22A18, SLC22A18AS, SLC22A2, SLC22A3, SLC22A4, SLC22A5, SLC22A6, SLC22A7, SLC22A8, SLC22A9, SLC27A1, SLC28A1, SLC28A2, SLC28A3, SLC29A1, SLC29A2, SLC2A4, SLC2A5, SLC5A6, SLC6A6, SLC7A5, SLC7A7, SLC7A8, SLCO1A2, SLCO1B1, SLCO1B3, SLCO1C1, SLCO2A1, SLCO2B1, SLCO3A1, SLCO4A1, SLCO4C1, SLCO5A1, SLCO6A1, SOD1, SOD2, SOD3, SULF1, SULT1A1, SULT1A2, SULT1A3, SULT1B1, SULT1C2, SULT1C4, SULT1E1, SULT2A1, SULT2B1, SULT4A1, TAP1, TAP2, TPMT, UGT1A1, UGT1A10, UGT1A3, UGT1A4, UGT1A5, UGT1A6, UGT1A7, UGT1A8, UGT1A9, UGT2A1, UGT2B10, UGT2B11, UGT2B15, UGT2B17, UGT2B28, UGT2B4, UGT2B7, UGT8, XDH, DRD2, VKORC1* |
| CPIC VIPs | *ABCB1, ABCG2, ACE, ADRB1, ADRB2, CACNA1S, CFTR, COMT, CYP2A6, CYP2B6, CYP2C19, CYP2C8, CYP2C9, CYP2D6, CYP3A4, CYP3A5, CYP4F2, DPYD, DRD2, F5, G6PD, GSTT4, HLA-B, MTHFR, NAT2, NUDT15, RYR1, SLC19A1, SLCO1B1, TPMT, TYMS, UGT1A1, VKORC1, ADH1A, ADH1B, ADH1C, AHR, ALDH1A1, ALOX5, CYP1A2, CYP2A13, CYP2E1, CYP2J2, GSTT1, HMGCR, KCNH2, KCNJ11, NQO1, NR1I2, P2RY1, P2RY12, PTGIS, PTGS2, SCN5A, SLC22A1, SULT1A1, VDR* |

**Supplementary Table 2**: GLMM regression coefficients for each of the gene sets and variant class combination analysed

| **Metabolite** | **Gene set analysed** | **Variant class tested** | **# alleles tested** | **Beta** | **SE** | **P** | **partR2** | **FDR P** |
| --- | --- | --- | --- | --- | --- | --- | --- | --- |
| clozapine | Clozapine pathway | PTVs + missense | 398 | -0.0310 | 0.0260 | 0.2337 | 0.149% | 0.4772 |
| clozapine | Clozapine pathway | missense | 368 | -0.0368 | 0.0268 | 0.1701 | 0.186% | 0.4535 |
| clozapine | Clozapine pathway | PTVs | 30 | 0.0512 | 0.0978 | 0.6003 | 0.001% | 0.8185 |
| clozapine | Clozapine pathway | synonymous | 246 | -0.0095 | 0.0344 | 0.7818 | 0.009% | 0.8934 |
| clozapine | PharmaADME core | PTVs + missense | 758 | -0.0542 | 0.0193 | 0.0049 | 0.407% | 0.0781 |
| clozapine | PharmaADME core | missense | 685 | -0.0479 | 0.0202 | 0.0174 | 0.322% | 0.1394 |
| clozapine | PharmaADME core | PTVs | 73 | -0.1123 | 0.0633 | 0.0759 | 0.107% | 0.3038 |
| clozapine | PharmaADME core | synonymous | 464 | -0.0027 | 0.0243 | 0.9128 | 0.003% | 0.9736 |
| clozapine | PharmaADME extended | PTVs + missense | 5449 | 0.0001 | 0.0072 | 0.9941 | 0.000% | 0.9941 |
| clozapine | PharmaADME extended | missense | 4872 | -0.0025 | 0.0076 | 0.7432 | 0.031% | 0.8934 |
| clozapine | PharmaADME extended | PTVs | 577 | 0.0211 | 0.0219 | 0.3360 | 0.000% | 0.5973 |
| clozapine | PharmaADME extended | synonymous | 4296 | 0.0065 | 0.0080 | 0.4123 | 0.009% | 0.6597 |
| clozapine | CPIC VIPs | PTVs + missense | 1403 | -0.0227 | 0.0136 | 0.0951 | 0.164% | 0.3045 |
| clozapine | CPIC VIPs | missense | 1280 | -0.0168 | 0.0143 | 0.2386 | 0.099% | 0.4772 |
| clozapine | CPIC VIPs | PTVs | 123 | -0.0929 | 0.0480 | 0.0529 | 0.141% | 0.2823 |
| clozapine | CPIC VIPs | synonymous | 1241 | -0.0075 | 0.0150 | 0.6139 | 0.004% | 0.8185 |
| norclozapine | Clozapine pathway | PTVs + missense | 398 | -0.0207 | 0.0240 | 0.3890 | 0.091% | 0.6360 |
| norclozapine | Clozapine pathway | missense | 368 | -0.0258 | 0.0247 | 0.2974 | 0.116% | 0.6360 |
| norclozapine | Clozapine pathway | PTVs | 30 | 0.0503 | 0.0900 | 0.5763 | 0.000% | 0.7684 |
| norclozapine | Clozapine pathway | synonymous | 246 | -0.0091 | 0.0317 | 0.7742 | 0.004% | 0.8613 |
| norclozapine | PharmaADME core | PTVs + missense | 758 | -0.0432 | 0.0178 | 0.0150 | 0.332% | 0.2399 |
| norclozapine | PharmaADME core | missense | 685 | -0.0361 | 0.0186 | 0.0517 | 0.234% | 0.2405 |
| norclozapine | PharmaADME core | PTVs | 73 | -0.1098 | 0.0584 | 0.0601 | 0.147% | 0.2405 |
| norclozapine | PharmaADME core | synonymous | 464 | -0.0039 | 0.0225 | 0.8613 | 0.003% | 0.8613 |
| norclozapine | PharmaADME extended | PTVs + missense | 5449 | -0.0054 | 0.0066 | 0.4196 | 0.095% | 0.6360 |
| norclozapine | PharmaADME extended | missense | 4872 | -0.0055 | 0.0070 | 0.4373 | 0.093% | 0.6360 |
| norclozapine | PharmaADME extended | PTVs | 577 | -0.0047 | 0.0202 | 0.8162 | 0.007% | 0.8613 |
| norclozapine | PharmaADME extended | synonymous | 4296 | 0.0085 | 0.0073 | 0.2442 | 0.043% | 0.6360 |
| norclozapine | CPIC VIPs | PTVs + missense | 1403 | -0.0110 | 0.0126 | 0.3792 | 0.075% | 0.6360 |
| norclozapine | CPIC VIPs | missense | 1280 | -0.0041 | 0.0132 | 0.7565 | 0.022% | 0.8613 |
| norclozapine | CPIC VIPs | PTVs | 123 | -0.0914 | 0.0442 | 0.0387 | 0.199% | 0.2405 |
| norclozapine | CPIC VIPs | synonymous | 1241 | -0.0133 | 0.0138 | 0.3348 | 0.000% | 0.6360 |

**Supplementary Table 3:** Effect sizes for each of the genetic, demographic, and clinical covariates included in the GLMM in the PharmaADME core gene set association with clozapine plasma concentrations

| **Predictor** | **β** | **SE** | **P** | **Component** |
| --- | --- | --- | --- | --- |
| PTVs+missense in PharmaADME core set | -0.0542 | 0.0193 | 4.88E-03 | mean |
| Daily dose (mg/day) | 0.0015 | 0.0001 | 3.12E-176 | mean |
| Time between dose and blood draw (hours) ⱡ | -0.0131 | 0.0059 | 2.57E-02 | mean |
| Sex (male) | -0.1108 | 0.0265 | 2.92E-05 | mean |
| Age (years) ⱡ | 0.0416 | 0.0126 | 1.01E-03 | mean |
| Age^2^ (years^2^) ⱡ | 0.0098 | 0.0112 | 3.80E-01 | mean |
| rs1126545_T | 0.0233 | 0.0238 | 3.26E-01 | mean |
| rs2472297_T | -0.0984 | 0.0186 | 1.27E-07 | mean |
| rs61750900_T | 0.0311 | 0.0278 | 2.64E-01 | mean |
| rs2011425_G | -0.1149 | 0.0300 | 1.31E-04 | mean |
| PC1 ⱡ | 0.0292 | 0.0136 | 3.18E-02 | mean |
| PC2 ⱡ | 0.0131 | 0.0115 | 2.57E-01 | mean |
| PC3 ⱡ | -0.0015 | 0.0116 | 8.95E-01 | mean |
| PC4 ⱡ | -0.0051 | 0.0115 | 6.56E-01 | mean |
| PC5 ⱡ | -0.0012 | 0.0117 | 9.19E-01 | mean |
| PC6 ⱡ | 0.0032 | 0.0114 | 7.81E-01 | mean |
| PC7 ⱡ | -0.0082 | 0.0115 | 4.74E-01 | mean |
| PC8 ⱡ | -0.0052 | 0.0117 | 6.55E-01 | mean |
| PC9 ⱡ | -0.0065 | 0.0114 | 5.71E-01 | mean |
| PC10 ⱡ | -0.0022 | 0.0117 | 8.52E-01 | mean |
| Synonymous variant count | -0.0001 | 0.0007 | 8.83E-01 | mean |
| Daily dose (mg/day) | 1.9444 | 0.0632 | 4.89E-208 | variance |
| Time between dose and blood draw (hours) ⱡ | 0.0008 | 0.0001 | 1.54E-09 | variance |
| Sex (male) | -0.0741 | 0.0218 | 6.71E-04 | variance |
| Age (years) ⱡ | -0.1213 | 0.0489 | 1.31E-02 | variance |
| Age^2^ (years^2^) ⱡ | 0.0447 | 0.0222 | 4.42E-02 | variance |

Effect size coefficients (β) indicate the positive or negative impact of a one-unit increase of the predictor in the average of clozapine concentrations in ng/mL. Predictors marked with ⱡ were standardised to improve model fit and their effect sizes refer to a one standard deviation increase(Gelman et al., 2020).

**Supplementary Table 4:** Individual gene level association with clozapine plasma concentrations using the same GLMM framework. Only genes within the PharmaADME core list and presenting at least 5 rare damaging alleles were analysed.

| **Metabolite** | **Gene** | **Number alleles tested** | **Beta** | **SE** | **partR2** | **P** |
| --- | --- | --- | --- | --- | --- | --- |
| clozapine | *CYP1A2* | 17 (1 PTVs + 16 missense) | 0.3242 | 0.1248 | 0.233% | 0.0094 |
| clozapine | *UGT1A1* | 19 (0 PTVs + 19 missense) | -0.2585 | 0.1221 | 0.144% | 0.0342 |
| clozapine | *CYP1A1* | 63 (5 PTVs + 58 missense) | -0.1286 | 0.0660 | 0.145% | 0.0514 |
| clozapine | *DPYD* | 90 (25 PTVs + 65 missense) | -0.1033 | 0.0564 | 0.113% | 0.0672 |
| clozapine | *CYP3A4* | 44 (4 PTVs + 40 missense) | -0.1468 | 0.0812 | 0.141% | 0.0705 |
| clozapine | *CYP2D6* | 14 (8 PTVs + 6 missense) | 0.2598 | 0.1448 | 0.022% | 0.0728 |
| clozapine | *SLCO1B1* | 8 (1 PTVs + 7 missense) | -0.3378 | 0.1919 | 0.099% | 0.0784 |
| clozapine | *SULT1A1* | 12 (1 PTVs + 11 missense) | 0.2517 | 0.1494 | 0.047% | 0.0920 |
| clozapine | *SLCO1B3* | 6 (1 PTVs + 5 missense) | -0.2794 | 0.2183 | 0.058% | 0.2005 |
| clozapine | *ABCB1* | 40 (2 PTVs + 38 missense) | -0.1060 | 0.0835 | 0.078% | 0.2044 |
| clozapine | *CYP2C9* | 38 (0 PTVs + 38 missense) | -0.1070 | 0.0900 | 0.068% | 0.2344 |
| clozapine | *SLC22A6* | 24 (0 PTVs + 24 missense) | -0.1283 | 0.1114 | 0.026% | 0.2493 |
| clozapine | *CYP2B6* | 33 (0 PTVs + 33 missense) | -0.0962 | 0.0921 | 0.037% | 0.2964 |
| clozapine | *TPMT* | 13 (1 PTVs + 12 missense) | 0.1368 | 0.1460 | 0.015% | 0.3491 |
| clozapine | *SLC22A2* | 6 (0 PTVs + 6 missense) | -0.1921 | 0.2220 | 0.018% | 0.3867 |
| clozapine | *UGT2B7* | 16 (0 PTVs + 16 missense) | -0.1125 | 0.1322 | 0.042% | 0.3948 |
| clozapine | *ABCG2* | 21 (2 PTVs + 19 missense) | -0.0797 | 0.1141 | 0.024% | 0.4848 |
| clozapine | *CYP2C8* | 32 (3 PTVs + 29 missense) | -0.0566 | 0.0957 | 0.028% | 0.5540 |
| clozapine | *GSTP1* | 8 (1 PTVs + 7 missense) | 0.0979 | 0.1808 | 0.008% | 0.5883 |
| clozapine | *SLC6A2* | 14 (0 PTVs + 14 missense) | -0.0733 | 0.1437 | 0.011% | 0.6102 |
| clozapine | *UGT2B15* | 26 (1 PTVs + 25 missense) | -0.0392 | 0.1017 | 0.000% | 0.7002 |
| clozapine | *SLC22A1* | 40 (0 PTVs + 40 missense) | 0.0145 | 0.0845 | 0.000% | 0.8640 |
| clozapine | *SLC15A2* | 15 (1 PTVs + 14 missense) | 0.0227 | 0.1375 | 0.007% | 0.8687 |
| clozapine | *CYP3A5* | 17 (8 PTVs + 9 missense) | 0.0163 | 0.1256 | 0.000% | 0.8964 |
| clozapine | *ABCC2* | 116 (7 PTVs + 109 missense) | -0.0060 | 0.0501 | 0.006% | 0.9046 |
| clozapine | *CYP2C19* | 22 (0 PTVs + 22 missense) | 0.0033 | 0.1106 | 0.005% | 0.9759 |

**Supplementary Table 5**: Individual gene level association with norclozapine plasma concentrations using the same GLMM framework. Only genes within the PharmaADME core list and presenting at least 5 rare damaging alleles were analysed.

| **Metabolite** | **Gene** | **Number alleles tested** | **Beta** | **SE** | **partR2** | **P** |
| --- | --- | --- | --- | --- | --- | --- |
| norclozapine | *CYP1A2* | 17 (1 PTVs + 16 missense) | 0.3201 | 0.1151 | 0.235% | 0.0054 |
| norclozapine | *SULT1A1* | 12 (1 PTVs + 11 missense) | 0.2816 | 0.1379 | 0.036% | 0.0412 |
| norclozapine | *DPYD* | 90 (25 PTVs + 65 missense) | -0.0846 | 0.0520 | 0.106% | 0.1037 |
| norclozapine | *CYP1A1* | 63 (5 PTVs + 58 missense) | -0.0967 | 0.0608 | 0.092% | 0.1119 |
| norclozapine | *UGT1A1* | 19 (0 PTVs + 19 missense) | -0.1700 | 0.1130 | 0.096% | 0.1327 |
| norclozapine | *CYP3A4* | 44 (4 PTVs + 40 missense) | -0.1016 | 0.0747 | 0.082% | 0.1738 |
| norclozapine | *SLC22A2* | 6 (0 PTVs + 6 missense) | -0.2507 | 0.2048 | 0.020% | 0.2210 |
| norclozapine | *ABCB1* | 40 (2 PTVs + 38 missense) | -0.0902 | 0.0770 | 0.089% | 0.2411 |
| norclozapine | *CYP2D6* | 14 (8 PTVs + 6 missense) | 0.1511 | 0.1340 | 0.000% | 0.2596 |
| norclozapine | *SLCO1B1* | 8 (1 PTVs + 7 missense) | -0.1982 | 0.1764 | 0.048% | 0.2611 |
| norclozapine | *UGT2B7* | 16 (0 PTVs + 16 missense) | -0.1174 | 0.1216 | 0.051% | 0.3342 |
| norclozapine | *SLC22A6* | 24 (0 PTVs + 24 missense) | -0.0920 | 0.1024 | 0.015% | 0.3693 |
| norclozapine | *ABCG2* | 21 (2 PTVs + 19 missense) | -0.0939 | 0.1049 | 0.032% | 0.3708 |
| norclozapine | *CYP2B6* | 33 (0 PTVs + 33 missense) | -0.0720 | 0.0853 | 0.024% | 0.3990 |
| norclozapine | *SLC6A2* | 14 (0 PTVs + 14 missense) | -0.1089 | 0.1323 | 0.016% | 0.4106 |
| norclozapine | *SLC22A1* | 40 (0 PTVs + 40 missense) | 0.0591 | 0.0779 | 0.000% | 0.4479 |
| norclozapine | *SLCO1B3* | 6 (1 PTVs + 5 missense) | -0.1434 | 0.2012 | 0.015% | 0.4761 |
| norclozapine | *UGT2B15* | 26 (1 PTVs + 25 missense) | -0.0531 | 0.0938 | 0.000% | 0.5718 |
| norclozapine | *CYP2C9* | 38 (0 PTVs + 38 missense) | -0.0456 | 0.0831 | 0.012% | 0.5837 |
| norclozapine | *TPMT* | 13 (1 PTVs + 12 missense) | 0.0671 | 0.1346 | 0.006% | 0.6181 |
| norclozapine | *CYP2C8* | 32 (3 PTVs + 29 missense) | -0.0391 | 0.0877 | 0.023% | 0.6557 |
| norclozapine | *CYP2C19* | 22 (0 PTVs + 22 missense) | -0.0316 | 0.1023 | 0.015% | 0.7571 |
| norclozapine | *SLC15A2* | 15 (1 PTVs + 14 missense) | -0.0368 | 0.1266 | 0.000% | 0.7715 |
| norclozapine | *GSTP1* | 8 (1 PTVs + 7 missense) | 0.0454 | 0.1667 | 0.005% | 0.7855 |
| norclozapine | *ABCC2* | 116 (7 PTVs + 109 missense) | -0.0120 | 0.0462 | 0.012% | 0.7955 |
| norclozapine | *CYP3A5* | 17 (8 PTVs + 9 missense) | 0.0199 | 0.1157 | 0.000% | 0.8635 |

**Supplementary Table 6**: Effect sizes for each of the covariates included in the GLMM in the CYP1A2 association with clozapine plasma concentrations.

| **Predictor** | **Beta** | **SE** | **P** | **Component** |
| --- | --- | --- | --- | --- |
| *CYP1A2* | 0.3242 | 0.1248 | 9.40E-03 | mean |
| Daily dose (mg/day) | 0.0015 | 0.0001 | 4.24E-178 | mean |
| Time between dose and blood draw (hours) ⱡ | 0.0440 | 0.0126 | 5.01E-04 | mean |
| Sex (male) | -0.1126 | 0.0265 | 2.17E-05 | mean |
| Age (years) ⱡ | 0.0096 | 0.0112 | 3.92E-01 | mean |
| Age2 (years2) ⱡ | -0.0131 | 0.0059 | 2.61E-02 | mean |
| rs1126545_T | 0.0215 | 0.0238 | 3.67E-01 | mean |
| rs2472297_T | -0.0980 | 0.0186 | 1.47E-07 | mean |
| rs61750900_T | 0.0289 | 0.0278 | 2.98E-01 | mean |
| rs2011425_G | -0.1222 | 0.0300 | 4.72E-05 | mean |
| PC1 ⱡ | 0.0311 | 0.0136 | 2.25E-02 | mean |
| PC2 ⱡ | 0.0131 | 0.0115 | 2.57E-01 | mean |
| PC3 ⱡ | -0.0013 | 0.0116 | 9.13E-01 | mean |
| PC4 ⱡ | -0.0063 | 0.0115 | 5.82E-01 | mean |
| PC5 ⱡ | 0.0006 | 0.0117 | 9.57E-01 | mean |
| PC6 ⱡ | 0.0040 | 0.0115 | 7.25E-01 | mean |
| PC7 ⱡ | -0.0075 | 0.0115 | 5.15E-01 | mean |
| PC8 ⱡ | -0.0043 | 0.0117 | 7.13E-01 | mean |
| PC9 ⱡ | -0.0056 | 0.0114 | 6.24E-01 | mean |
| PC10 ⱡ | -0.0030 | 0.0117 | 8.00E-01 | mean |
| Synonymous variant count | -0.0001 | 0.0007 | 8.71E-01 | mean |
| Daily dose (mg/day) | 0.0008 | 0.0001 | 1.55E-09 | variance |
| Time between dose and blood draw (hours) ⱡ | 0.0443 | 0.0222 | 4.59E-02 | variance |
| Sex (male) | -0.1209 | 0.0489 | 1.35E-02 | variance |
| Age (years) ⱡ | 0.0095 | 0.0201 | 6.38E-01 | variance |
| Age2 (years2) ⱡ | -0.0744 | 0.0218 | 6.34E-04 | variance |

Effect size coefficients (Beta) are given in a ng/mL scale. Predictors marked with ⱡ were standardised to improve model fit and their effect sizes refer to a one standard deviation increase.

**Supplementary Table 7**: CYP1A2 rare damaging variants observed in CLOZUK2.

| **CHR** | **BP GRCh37** | **rsid** | **Ref** | **Alter** | **MAC** | **MAF** | **Variant type** | **CADD PHRED-score** | **MAC**  **NFE UKBB(Karczewski et al., 2022)** | **PharmGKB annotation** |
| --- | --- | --- | --- | --- | --- | --- | --- | --- | --- | --- |
| 15 | 75042389 | rs34067076 | G | A | 2 | 0.000486 | missense | 24.6 | 540 | NA |
| 15 | 75042492 | rs59410695 | G | A | 1 | 0.000243 | missense | 23.7 | 147 | NA |
| 15 | 75042792 | rs200571120 | T | C | 1 | 0.000242 | missense | 25.5 | NA | NA |
| 15 | 75043539 | rs45468096 | C | T | 6 | 0.001456 | missense | 25.4 | 1408 | NA |
| 15 | 75044578 | rs72547516 | A | G | 5 | 0.001214 | missense | 22.1 | 682 | Allele T corresponds to CYP1A2*4 (note, here, however, we observe allele G). Allele T is associated with decreased metabolism of clozapine as compared to allele A. (https://www.pharmgkb.org/variantAnnotation/699638724) |
| 15 | 75047218 | rs566851431 | A | T | 1 | 0.000244 | missense | 24.5 | NA | NA |
| 15 | 75047348 | . | C | CCTGA | 1 | 0.000245 | insertion/  frameshift |  | NA | NA |

**Supplementary Table 8**: GLMM coefficients for the effect of the significant gene sets on the distributional regression models

|  |  |  | **Mean** | | | **Variance** | | |
| --- | --- | --- | --- | --- | --- | --- | --- | --- |
| **Metabolite** | **Gene set analysed** | **Variant class tested** | **Beta** | **SE** | **P** | **disp.Beta** | **disp.SE** | **disp.P** |
| clozapine | PharmaADME core | PTVs + missense | -0.0538 | 0.0193 | 0.0053 | -0.0122 | 0.0353 | 0.7298 |
| clozapine | PharmaADME core | missense | -0.0466 | 0.0202 | 0.0215 | -0.0439 | 0.0365 | 0.2290 |
| clozapine | PharmaADME core | PTVs | -0.1237 | 0.0612 | 0.0430 | 0.3959 | 0.1187 | 0.0009 |
| norclozapine | PharmaADME core | PTVs + missense | -0.0439 | 0.0177 | 0.0132 | 0.0277 | 0.0356 | 0.4358 |
| norclozapine | PharmaADME core | missense | -0.0361 | 0.0186 | 0.0520 | -0.0013 | 0.0368 | 0.9728 |
| norclozapine | PharmaADME core | PTVs | -0.1191 | 0.0565 | 0.0351 | 0.3686 | 0.1179 | 0.0018 |

## References

Brooks, M.E., Kristensen, K., van Benthem, K.J., Magnusson, A., Berg, C.W., Nielsen, A., Skaug, H.J., Machler, M., Bolker, B.M., 2017. glmmTMB Balances Speed and Flexibility Among Packages for Zero-inflated Generalized Linear Mixed Modeling. R J 9, 378-400.

Conomos, M.P., Reiner, A.P., Weir, B.S., Thornton, T.A., 2016. Model-free Estimation of Recent Genetic Relatedness. Am J Hum Genet 98, 127-148.

de Villemereuil, P., Morrissey, M.B., Nakagawa, S., Schielzeth, H., 2018. Fixed-effect variance and the estimation of repeatabilities and heritabilities: issues and solutions. J Evolution Biol 31, 621-632.

Diaz, F.J., de Leon, J., Josiassen, R.C., Cooper, T.B., Simpson, G.M., 2005. Plasma clozapine concentration coefficients of variation in a long-term study. Schizophrenia Research 72, 131-135.

Gelman, A., Hill, J., Vehtari, A., 2020. Regression and Other Stories. Cambridge University Press.

Genomes Project, C., Auton, A., Brooks, L.D., Durbin, R.M., Garrison, E.P., Kang, H.M., Korbel, J.O., Marchini, J.L., McCarthy, S., McVean, G.A., Abecasis, G.R., 2015. A global reference for human genetic variation. Nature 526, 68-74.

Hail_Team, Hail 0.2, <https://github.com/hail-is/hail>.

Jakobsen, M.I., Larsen, J.R., Svensson, C.K., Johansen, S.S., Linnet, K., Nielsen, J., Fink-Jensen, A., 2017. The significance of sampling time in therapeutic drug monitoring of clozapine. Acta Psychiatrica Scandinavica 135, 159-169.

Karczewski, K.J., Francioli, L.C., Tiao, G., Cummings, B.B., Alfoldi, J., Wang, Q., Collins, R.L., Laricchia, K.M., Ganna, A., Birnbaum, D.P., Gauthier, L.D., Brand, H., Solomonson, M., Watts, N.A., Rhodes, D., Singer-Berk, M., England, E.M., Seaby, E.G., Kosmicki, J.A., Walters, R.K., Tashman, K., Farjoun, Y., Banks, E., Poterba, T., Wang, A., Seed, C., Whiffin, N., Chong, J.X., Samocha, K.E., Pierce-Hoffman, E., Zappala, Z., O'Donnell-Luria, A.H., Minikel, E.V., Weisburd, B., Lek, M., Ware, J.S., Vittal, C., Armean, I.M., Bergelson, L., Cibulskis, K., Connolly, K.M., Covarrubias, M., Donnelly, S., Ferriera, S., Gabriel, S., Gentry, J., Gupta, N., Jeandet, T., Kaplan, D., Llanwarne, C., Munshi, R., Novod, S., Petrillo, N., Roazen, D., Ruano-Rubio, V., Saltzman, A., Schleicher, M., Soto, J., Tibbetts, K., Tolonen, C., Wade, G., Talkowski, M.E., Genome Aggregation Database, C., Neale, B.M., Daly, M.J., MacArthur, D.G., 2020. The mutational constraint spectrum quantified from variation in 141,456 humans. Nature 581, 434-443.

Karczewski, K.J., Solomonson, M., Chao, K.R., Goodrich, J.K., Tiao, G., Lu, W., Riley-Gillis, B.M., Tsai, E.A., Kim, H.I., Zheng, X., Rahimov, F., Esmaeeli, S., Grundstad, A.J., Reppell, M., Waring, J., Jacob, H., Sexton, D., Bronson, P.G., Chen, X., Hu, X., Goldstein, J.I., King, D., Vittal, C., Poterba, T., Palmer, D.S., Churchhouse, C., Howrigan, D.P., Zhou, W., Watts, N.A., Nguyen, K., Nguyen, H., Mason, C., Farnham, C., Tolonen, C., Gauthier, L.D., Gupta, N., MacArthur, D.G., Rehm, H.L., Seed, C., Philippakis, A.A., Daly, M.J., Davis, J.W., Runz, H., Miller, M.R., Neale, B.M., 2022. Systematic single-variant and gene-based association testing of thousands of phenotypes in 394,841 UK Biobank exomes. Cell Genom 2, 100168.

Kneib, T., Silbersdorff, A., Säfken, B., 2021. Rage Against the Mean – A Review of Distributional Regression Approaches. Econometrics and Statistics.

Li, H., Durbin, R., 2009. Fast and accurate short read alignment with Burrows-Wheeler transform. Bioinformatics 25, 1754-1760.

McKenna, A., Hanna, M., Banks, E., Sivachenko, A., Cibulskis, K., Kernytsky, A., Garimella, K., Altshuler, D., Gabriel, S., Daly, M., DePristo, M.A., 2010. The Genome Analysis Toolkit: a MapReduce framework for analyzing next-generation DNA sequencing data. Genome Res 20, 1297-1303.

Pedersen, B.S., Quinlan, A.R., 2017. Who's Who? Detecting and Resolving Sample Anomalies in Human DNA Sequencing Studies with Peddy. Am J Hum Genet 100, 406-413.

Rentzsch, P., Schubach, M., Shendure, J., Kircher, M., 2021. CADD-Splice-improving genome-wide variant effect prediction using deep learning-derived splice scores. Genome Med 13, 31.

Rentzsch, P., Witten, D., Cooper, G.M., Shendure, J., Kircher, M., 2019. CADD: predicting the deleteriousness of variants throughout the human genome. Nucleic acids research 47, D886-D894.

Roberson, Q.M., Sturman, M.C., Simons, T.L., 2007. Does the Measure of Dispersion Matter in Multilevel Research? A Comparison of the Relative Performance of Dispersion Indexes. Organizational Research Methods 10, 564-588.

Walters, R.W., Hoffman, L., Templin, J., 2018. The Power to Detect and Predict Individual Differences in Intra-Individual Variability Using the Mixed-Effects Location-Scale Model. Multivariate Behavioral Research 53, 360-374.
